# A New Dimension of Health Care: Systematic Review of the Uses, Benefits, and Limitations of Social Media for Health Communication

**DOI:** 10.2196/jmir.1933

**Published:** 2013-04-23

**Authors:** S Anne Moorhead, Diane E Hazlett, Laura Harrison, Jennifer K Carroll, Anthea Irwin, Ciska Hoving

**Affiliations:** ^1^School of CommunicationUniversity of UlsterNewtownabbey, Northern IrelandUnited Kingdom; ^2^Family Medicine Research ProgramsDepartment of Family MedicineUniversity of Rochester Medical CenterRochester, NYUnited States; ^3^CAPHRI School for Public Health and Primary Care, Department of Health PromotionMaastricht UniversityMaastrichtNetherlands

**Keywords:** health communication, social media, review

## Abstract

**Background:**

There is currently a lack of information about the uses, benefits, and limitations of social media for health communication among the general public, patients, and health professionals from primary research.

**Objective:**

To review the current published literature to identify the uses, benefits, and limitations of social media for health communication among the general public, patients, and health professionals, and identify current gaps in the literature to provide recommendations for future health communication research.

**Methods:**

This paper is a review using a systematic approach. A systematic search of the literature was conducted using nine electronic databases and manual searches to locate peer-reviewed studies published between January 2002 and February 2012.

**Results:**

The search identified 98 original research studies that included the uses, benefits, and/or limitations of social media for health communication among the general public, patients, and health professionals. The methodological quality of the studies assessed using the Downs and Black instrument was low; this was mainly due to the fact that the vast majority of the studies in this review included limited methodologies and was mainly exploratory and descriptive in nature. Seven main uses of social media for health communication were identified, including focusing on increasing interactions with others, and facilitating, sharing, and obtaining health messages. The six key overarching benefits were identified as (1) increased interactions with others, (2) more available, shared, and tailored information, (3) increased accessibility and widening access to health information, (4) peer/social/emotional support, (5) public health surveillance, and (6) potential to influence health policy. Twelve limitations were identified, primarily consisting of quality concerns and lack of reliability, confidentiality, and privacy.

**Conclusions:**

Social media brings a new dimension to health care as it offers a medium to be used by the public, patients, and health professionals to communicate about health issues with the possibility of potentially improving health outcomes. Social media is a powerful tool, which offers collaboration between users and is a social interaction mechanism for a range of individuals. Although there are several benefits to the use of social media for health communication, the information exchanged needs to be monitored for quality and reliability, and the users’ confidentiality and privacy need to be maintained. Eight gaps in the literature and key recommendations for future health communication research were provided. Examples of these recommendations include the need to determine the relative effectiveness of different types of social media for health communication using randomized control trials and to explore potential mechanisms for monitoring and enhancing the quality and reliability of health communication using social media. Further robust and comprehensive evaluation and review, using a range of methodologies, are required to establish whether social media improves health communication practice both in the short and long terms.

## Introduction

There is an ongoing increase in the use of social media globally [1], including in health care contexts [2-9]. When focusing on social media for health communication, it is useful to first outline the general characteristics of social media. Kaplan and Haenlein [10] defined social media as “a group of Internet-based applications that build on the ideological and technological foundations of Web 2.0, and that allow the creation and exchange of user generated content”. They suggested that social media can be classified as two components: media-related and social dimension. The media-related component [11] involves how close to synchronous face-to-face communication different types of social media come and how well they reduce ambiguity and uncertainty. The social dimension is based on Goffman’s [12] notion of self-presentation, whereby individuals’ interactions have the purpose of trying to control others’ impressions of them.

Social media provides opportunities for users to generate, share, receive, and comment on social content among multiusers through multisensory communication [1,2,10,13,14]. Although the terms “social media” and “social networking” are often used interchangeably and have some overlaps, they are not really the same. Social media functions as a communication channel that delivers a message, which involves asking for something. Social networking is two-way and direct communication that includes sharing of information between several parties. Social media can be classified in a number of ways to reflect the diverse range of social media platforms, such as collaborative projects (eg, Wikipedia), content communities (eg, YouTube), social networking sites (eg, Facebook), and virtual game and social worlds (eg, World of Warcraft, Second Life) [10].

The relationship between personality traits and engagement with social media has been reported [15]. Gender is a factor in that extraverted women and men are equally likely to engage, but emotional instability increases usage only for men. Age is also a factor in that extraversion is particularly important in younger users, while openness to new experiences is particularly important in older users [15]. Lenhart and colleagues [16] explored various types of Internet usage among teens and young adults in the United States between 2006 and 2010. During this time, social networking sites experienced the biggest rise (an average of around 50%), and the key shift in use came at age 30 years with almost double the number of teens and 18-29 years old using them as those 30 years and over (73% compared with 39%).

Social media is changing the nature and speed of health care interaction between individuals and health organizations. The general public, patients, and health professionals are using social media to communicate about health issues [2-9]. In the United States, 61% of adults search online and 39% use social media such as Facebook for health information [7]. Social media adoption rates vary in Europe; for example, the percentage of German hospitals using social networks is in “single figures”, whereas approximately 45% of Norwegian and Swedish hospitals are using LinkedIn, and 22% of Norwegian hospitals use Facebook for health communication [8]. Recent UK statistics reported Facebook as the fourth most popular source of health information [9]. There have been many applications of social media within health contexts, ranging from the World Health Organization using Twitter during the influenza A (H1N1) pandemic, with more than 11,700 followers [4], to medical practices [3] and health professionals obtaining information to inform their clinical practice [5,6].

To explore the diversity in form and function of different social media platforms, Keitzmann and colleagues [17] presented the “social media ecology”, a honeycomb framework of seven building blocks that are configured by different social media platforms and have different implications for organizations such as health care providers. In developing their model, they have drawn on Butterfield [18], Morville [19], Webb [20], and Smith [21]. The building blocks are (1) identity: the extent to which users reveal themselves, (2) conversations: the extent to which users communicate with each other, (3) sharing: the extent to which users exchange, distribute, and receive content, (4) presence: the extent to which users know if others are available, (5) relationships: the extent to which users relate to each other, (6) reputation: the extent to which users know the social standing of others and content, and (7) groups: the extent to which users are ordered or form communities. Thus organizations, including health care providers, need to recognize and understand the social media landscape, where the conversations about them are already being held, and develop their own strategies where suitable [17]. Similarly, Mangold and Faulds [22] highlighted that social media is changing the relationship between producers and consumers of a message. This suggests that health care providers may need to take a certain degree of control over online health communication to maintain validity and reliability.

In this paper, social media for health communication refers to the general public, patients, and health professionals communicating about health issues using social media platforms such as Facebook and Twitter. Currently, there is a lack of information about the uses, benefits, and limitations of social media for health communication among the general public, patients, and health professionals from primary research. The objective of this paper was to review the current published literature to identify the uses, benefits, and limitations of social media for health communication among the general public, patients, and health professionals and to identify current gaps in the literature to provide recommendations for future health communication research. This is important in order to establish whether social media improves health communication practices.

## Methods

**Figure 1:**
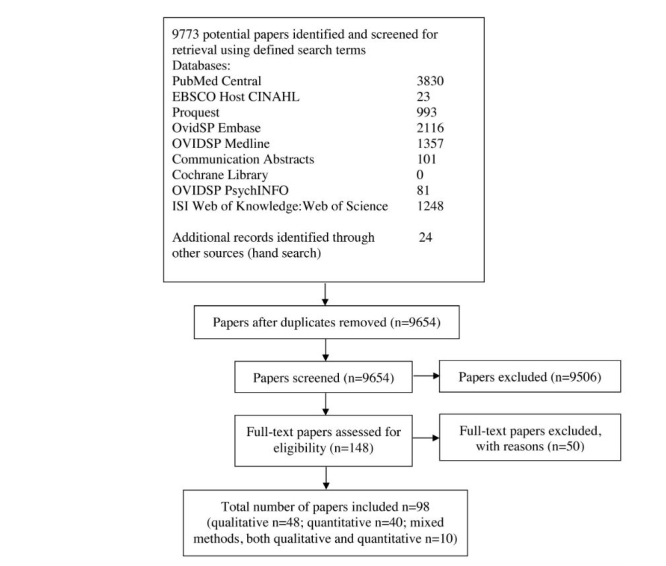
PRISMA flow diagram illustrating the study selection procedure.

This review paper followed the PRISMA guidelines [23] and used a systematic approach to retrieve the relevant research studies. The review included all study designs in order to identify the best evidence available to address the research objective. The literature search was conducted on February 7, 2012, using the following 10 electronic databases: CSA Illumina, Cochrane Library, Communication Abstracts, EBSCO Host CINAHL, ISI Web of Knowledge, Web of Science, OvidSP Embase, OvidSP MEDLINE, OVIDSP PsycINFO, and PubMeb Central. The searches were performed using the following defined search terms: “social media” OR “social network” OR “social networking” OR “Web 2.0” OR “Facebook” OR “Twitter” OR “MySpace” AND “Health”. From the above database searches, 9749 hits were identified. Manual searches were conducted in the *Journal of Medical Internet Research* (January 2002 to February 2012) where 24 papers were identified; thus, 9773 papers were identified in total. The papers’ titles and abstracts were screened for relevance, duplication, and the selection criteria. The inclusion criteria were (1) primary focus on all communication interactions within and between the general public and/or patients and/or health professionals about health issues using social media, (2) including the uses and/or benefits and/or limitations of social media for health communication, (3) original research studies, (4) published between January 2002 and February 2012, and (5) all study designs. The exclusion criteria were (1) studies not in English, (2) literature reviews, dissertation theses, review papers, reports, conference papers or abstracts, letters (to the editor), commentaries and feature articles, (3) studies only on Web 1.0 ( ie, traditional Internet use), and (4) studies with a primary marketing or advertising focus. In total, 98 original research studies that included the use, and/or benefits, and/or limitations of social media for health communication among the general public, patients, and health professionals were selected for this review [24-121] (see Figure 1). Excluded studies and the reasons for exclusion are listed in Multimedia Appendix 1. Two researchers (AM, LH) independently reviewed and evaluated the studies and reached consensus on the inclusion for the analysis. The interrater reliability between them was 0.90, indicating strong agreement [122]. Any discrepancies were discussed with reference to the research objective until consensus was reached.

## Results

The 98 selected studies are summarized by study design, social media tool/application, study purpose, participants/sample and sample size, measurement tools, results, conclusion, and use of social media in Multimedia Appendix 2 [24-121]. The diverse studies included the use of a range of social media tools/applications, the most reported being Facebook, blogs, Twitter, and YouTube (the full list is provided in Table 1 **)**. The study samples included blogs/forum discussions in which the participants were the general public, patients, and/or health professionals (Multimedia Appendix 2). There was a wide range of health topics, but the most frequently reported on were sexual health [45,46,70,104,107,115,117], diabetes [47,60,68,110,116], flu/H1N1 [54,57,58,111], and mental health issues such as stress or depression [48,83,101].

**Table 1:** Social media tools/applications within the 98 studies^a^.

Facebook (n=13)	Farmer et al (2009) [37], Ahmed et al (2010) [51], Greene et al (2010) [60], Bender et al (2011) [78], Egan & Moreno (2011a) [83], Egan & Moreno (2011b) [84], Frimmings et al (2011) [86], Gajara et al (2011)[88], Garcia-Romero et al (2011) [89], Jent et al (2011) [92], Kukreja et al (2011) [95], Lord et al (2011) [99], Sajadi & Goldman (2011) [105]
Blogs (n=13)	Adams (2008) [26], Kovic et al (2008) [29], Lagu et al (2008) [30], Tan (2008) [32], Denecke & Nedjl (2009) [36], Keelan et al (2009) [41], Kim (2009) [42], Adams (2010) [50], Clauson et al (2010) [53], Hu & Sundar (2010) [61], Sanford (2010) [71], Shah & Robinson (2011) [109], Marcus et al (2012) [119]
Twitter (n=8)	Chew & Eysenbach (2010) [54], Scanfeld et al (2010) [72], Heavillin et al (2011) [91], Kukreja et al (2011) [95], Sajadi & Goldman (2011) [105], Salathe & Khandelwal (2011) [106], Signorini et al (2011) [111], Turner-McGrievy & Tate (2011) [112]
YouTube (n=7)	Freeman & Chapman (2007) [24], Fernandez-Luque et al (2009) [38], Lo et al (2010) [67], Tian (2010) [74], Chou et al (2011) [80], Sajadi & Goldman (2011) [105], Fernandez-Luque et al (2012) [118]
MySpace (n=5)	Moreno et al (2007) [25], Moreno et al (2009a) [44], Moreno et al (2009b) [45], Versteeg et al (2009) [49], Ralph et al (2011) [104]
PatientsLikeMe (n=4)	Frost et al (2008) [28], Wicks et al (2010) [75], Doing-Harris & Zeng Treitler (2011) [81], Frost et al (2011) [87]
Wikipedia (n=3)	Clauson et al (2008) [27], Morturu & Liu (2011) [100], Rajagopalan et al (2011) [103]
Wiki (n=2)	Denecke & Nedjl (2009) [36], Adams (2010) [50]
Quitnet / online smoking cessation support group (n=2)	Cobb et al (2010) [55], Selby et al (2010) [73]
Physician rating website (not specified) (n=2)	Lagu (2010) [65], Kadry et al (2011) [93]
Second Life (n=1)	Beard et al (2009) [34]
Daily Strength (n=1)	Morturu & Liu (2011) [100]
ArboAntwoord (n=1)	Rhebergen et al (2012) [121]
Social media (tool not specified) (n=30)	Chou et al (2009) [35], Jennings et al (2009) [40], Takahashi et al (2009) [48], Avery et al (2010) [52], Colineau & Paris (2010) [56], Corley et al (2010) [57], Ding & Zhang (2010) [58], Hwang et al (2010) [62], Kim & Kwon (2010) [63], Kontos et al (2010) [64], Lariscy et al (2010) [66], Orizio et al (2010) [69], Rice et al (2010) [70], Adrie et al (2011) [76], Baptist et al (2011) [77], Bosslett et al (2011) [79], Dowdell et al (2011) [82], Friedman et al (2011) [85], Hanson et al (2011) [90], Kishimoto & Fukushmima (2011), [94], Lariscy et al (2011) [96], Liang & Scammon (2011) [98], O’Dea & Campbell (2011) [101], Omurtag et al (2011) [102], Selkie et al (2011) [107], Setoyama et al (2011) [108], Shrank et al (2011) [110], Veinot et al (2011) [115], Weitzman et al (2011) [116], Young & Rice (2011) [117], O’Grady et al (2012) [120]
Web 2.0 application (not specified) (n=11)	Scotch et al (2008) [31], Timpka et al (2008) [33], Hughes et al (2009) [39], Lupianez-Villanueva et al (2009) [43], Moen et al (2009) [44], Nordqvist et al (2009) [47], Ekberg et al (2010) [59], Nordfeldt et al (2010) [68], Lau (2011) [97], Usher et al (2011) [113], Van Uden-Kraan (2011) [114]

^a^Some studies included more than one social media tool/application.

### Methodological Quality of Studies

From the searches between January 2002 and February 2012, the selected studies in this review were published from 2007 to 2012 with the vast majority in the last 2 years (Table 1). From the available methodology of bias tools/quality scales, the Downs and Black Instrument [123] has been previously identified as a recommended tool to evaluate the quality of both quantitative randomized and nonrandomized studies [124]. As there are no standard accepted quality scales for studies of proportions [125], only quantitative studies (including mixed methods) were evaluated. Using this Downs and Black instrument [123], the maximum total score that could be achieved was 32, but the scores of the studies in this review ranged from 3 [89] to 22 [121]. Overall, the studies scored low using this scale as they were mainly exploratory and descriptive with three intervention studies [43,112,121] and one randomized controlled trial (RCT) [45]. From the 98 studies, 40 were applied quantitative, 48 qualitative (including studies with content analysis presenting data with descriptive statistics), and 10 mixed methods (both quantitative and quantitative). These studies are presented by methodology in Table 2. Methodological bias of the selected studies using the Downs and Black instrument [123] is presented in Multimedia Appendix 3.

### Characteristics/Profile of Users Accessing Social Media for Health Communication

The characteristics of users of social media for health communication in the selected studies were diverse, covering a range of different population groups. The age of the social media users ranged from school children to older adults aged 65 years and up [24-121], but the majority of the reported ages were 11-34 years [25,35,45,46,64,77,104,107,119]. Some studies reported that there were more female than male users of social network sites [35,40,55,62,64]. A few studies found that social media users were disproportionately from lower-income households [35,64,72]. Studies within the United States reported that more social media users were African Americans than nonHispanic Whites [35,40]. Chou et al [35] concluded that the population is accessing social media regardless of education and race/ethnicity.

### Uses of Social Media for Health Communication

From the selected studies, seven key uses of social media for health communication were identified for the general public, patients, and health professionals (Table 3). Social media provided health information on a range of conditions to the general public [36,61,71,74,103], patients [47,63,71,75,98,103], and health professionals [36,47,98]. This communication can provide answers to medical questions [34,36,60]. Social media allows information to be presented in modes other than text and can bring health information to audiences with special needs; for example, videos can be used to supplement or replace text and can be useful when literacy is low [50]. A range of social media platforms can facilitate dialogue between patients and patients, and patients and health professionals [56,79,110]. Sites such as PatientsLikeMe enable patients to engage in dialogue with each other and share health information and advice including information on treatment and medication [28,75]. YouTube has been used by the general public to share health information on medications, symptoms, and diagnoses [38], and by patients to share personal cancer stories [80]. Blog sites create a space where individuals can access tailored resources [26] and provide health professionals with an opportunity to share information with patients and members of the public [30,56]. Facebook is being used by the general public, patients, carers, and health professionals to share their experience of disease management, exploration, and diagnosis [37]. Asthma groups are using MySpace to share health information, in particular personal stories and experiences [49,60]. Social media can be used to collect data on patient experiences and opinions such as physician’s performance [26,65,109].

Social media have been used for health promotion and health education [25,34,46,59,82,90,113,117] and for delivering a health intervention by providing social support/influence to promote smoking cessation and abstinence [55]. A study has shown that social media can reduce stigma about certain conditions such as epilepsy [67]. In addition, there were some opportunities for health professionals to have online consultations [88].

### Benefits of Social Media for Health Communication

Six overarching benefits of social media for health communication were identified for the general public, patients, and health professionals (Table 4). Social media users have the potential to increase the number of interactions and thus are provided with more available, shared, and tailored information. Social media can generate more available health information as users create and share medical information online [50]. Blog sites create a space where individuals can access tailored resources to deal with health issues [26]. Social media can widen access to those who may not easily access health information via traditional methods, such as younger people, ethnic minorities, and lower socioeconomic groups [35,64,66,83,84,86,99,104,107,115]. An important aspect of using social media for health communication is that it can provide valuable peer, social, and emotional support for the general public [37,43,44,47,48,51,56,73,101,108,114] and patients [28,33,34,44,47,48,56,60,62,68,71,76,88,98,120]. For example, social media can aid health behavior change such as smoking cessation [53,73], and PatientsLikeMe enables patients to communicate with other patients and share information about health issues [28]. Colineau and Paris [56] reported that people used health-related social networking sites to discuss sensitive issues and complex information with health professionals.

In public health surveillance, social media can provide communication in real time and at relatively low cost [31,40,54,57,72,111,116]. Social media can monitor public response to health issues [54], track and monitor disease outbreak [111], identify misinformation of health information [72], identify target areas for intervention efforts [106], and disseminate pertinent health information to targeted communities [57]. Health professionals can aggregate data about patient experiences from blogs and monitor public reaction to health issues [26,40]. Social media may have particular potential for risk communications as they can be used to disseminate personalized messages immediately thus making outreach more effective [58]. There is the potential that information on social media may contribute to health care policy making, as medical blogs are frequently viewed by mainstream media [29].

**Table 2:** List of studies by methodology—quantitative, qualitative, or both (n=98).

Quantitative (n=40)	Qualitative (n=48)	Mixed methods (n=10)
Kovic et al (2008) [29]	Freeman & Chapman (2007)^a^[24]	Clauson et al (2008) [27]
Chou et al (2009) [35]	Moreno et al (2007)^a^[25]	Timpka et al (2008) [33]
Moreno et al (2009a) [45]	Adams (2008) [26]	Hughes et al (2009) [39]
Avery et al (2010) [52]	Frost et al (2008) [28]	Jennings et al (2009) [40]
Chew & Eysenbach (2010) [54]	Lagu et al (2008)^a^[30]	Lupianez-Villanueva et al (2009) [43]
Cobb et al (2010) [55]	Scotch et al (2008) [31]	Takahashi et al (2009) [48]
Colineau & Paris (2010) [56]	Tan (2008) [32]	Hwang et al (2010) [62]
Hu & Sundar (2010) [61]	Beard et al (2009) [34]	Ralph et al (2011) [104]
Kim & Kwon (2010) [63]	Denecke & Nedjl (2009)^a^[36]	Selkie et al (2011) [107]
Kontos et al (2010) [64]	Farmer et al (2009)^a^[37]	O’Grady et al (2012) [120]
Lariscy et al (2010) [66]	Fernandez-Luque et al (2009)^a^[38]	
Lo et al (2010) [67]	Keelan et al (2009)^a^[41]	
Rice et al (2010) [70]	Kim (2009) [42]	
Wicks et al (2010) [75]	Moen et al (2009) [44]	
Adrie et al (2011) [76]	Moreno et al (2009b)^a^[46]	
Baptist et al (2011) [77]	Nordqvist et al (2009) [47]	
Bosslett et al (2011) [79]	Versteeg et al (2009)^a^[49]	
Dowdell et al (2011) [82]	Adams (2010) [50]	
Frimmings et al (2011) [86]	Ahmed et al (2010)^a^[51]	
Garcia-Romero et al (2011) [89]	Clauson et al (2010) [53]	
Hanson et al (2011) [90]	Corley et al (2010) [57]	
Jent et al (2011) [92]	Ding & Zhang (2010)^a^[58]	
Kadry et al (2011) [93]	Ekberg (2010) [59]	
Kishimoto & Fukushmima (2011) [94]	Greene et al (2010)^a^[60]	
Kukreja et al (2011) [95]	Lagu (2010)^a^[65]	
Lau (2011) [97]	Nordfeldt et al (2010) [68]	
Lord et al (2011) [99]	Orizio et al (2010)^a^[69]	
Morturu & Liu (2011) [100]	Sanford (2010) [71]	
O’Dea & Campbell (2011) [101]	Scanfeld et al (2010) [72]	
Omurtag et al (2011) [102]	Selby et al (2010)^a^[73]	
Rajagopalan et al (2011) [103]	Tian (2010) [74]	
Setoyama et al (2011) [108]	Bender et al (2011)^a^[78]	
Signorini et al (2011) [111]	Chou et al (2011)^b^[80]	
Turner-McGrievy & Tate (2011) [112]	Doing-Harris & Zeng-Treitler (2011) [81]	
Usher et al (2011) [113]	Egan & Moreno (2011a)^a^[83]	
Van Uden-Kraan (2011) [114]	Egan & Moreno (2011b)^a^[84]	
Weitzman et al (2011) [116]	Friedman et al (2011)^a^[85]	
Young & Rice (2011) [117]	Frost et al (2011) [87]	
Fernandez-Luque et al (2012) [118]	Gajaria et al (2011) [88]	
Rhebergen et al (2012) [121]	Heavillin et al (2011) [91]	
	Lariscy et al (2011) [96]	
	Liang & Scammon (2011) [98]	
	Sajadi & Goldman (2011) [105]	
	Salthe & Khandelwal (2011)^a^[106]	
	Shah & Robinson (2011) [109]	
	Shrank et al (2011)^a^[110]	
	Veinot et al (2011) [115]	
	Marcus et al (2012) [119]	

^a^ Qualitative study using content analysis with some findings reported as descriptive statistics.

^b^ Descriptive statistics.

### Limitations of Using Social Media for Health Communication

There were 12 limitations of social media for health communication (Table 5). The main recurring limitations of social media are quality concerns [26,39,42,44,47,50,69,85] and the lack of reliability of the health information [26,37,39,40,42,44,47,50,69,74,85,95]. The authors of websites are often unidentifiable, or there can be numerous authors, or the line between producer and audience is blurred [38,50,74]. Thus it is more difficult for individuals to discern the reliability of information found online [50,38]. Regulations may not facilitate health professionals to communicate with patients online, for example, email is not an official medical record and could be vulnerable to security breaches [68]. Policy reactions to address concerns include providing training in how to use and navigate social media technologies and validate accuracy of information found [39,66], or bringing more credible sites into the mainstream and making them fully accessible [39].

The large volume of information available through social media and the possibility for inaccuracies posted on these sites presents challenges when validating information [26]. Several studies highlighted concerns about privacy and confidentiality, data security, and the potential harms that emerge when personal data are indexed [38,44,47,50]. Social media users are often unaware of the risks of disclosing personal information online [26] and with communicating harmful or incorrect advice using social media [26,50]. As information is readily available, there is the potential of information overload for the user [50]. The general public may not know how to correctly apply information found online to their personal health situation [50]. There is the potential that adverse health consequences can result from information found on social media sites, for example, pro-smoking imagery [24]. In addition, there may be negative health risk behaviors displayed online, such as unsafe sexual behavior [45,46]. There is limited evidence that engaging in online communities positively impacts people’s health [56]. Health professionals may not often use social media to communicate with their patients [42]. There is also the possibility that social media may act as a deterrent for patients from visiting health professionals [42].

**Table 3:** Uses of social media for health communication among the general public, patients, and health professionals.

Uses of social media for health communication	Social media user		
	General Public	Patients	Health Professionals
Provide health information on a range of conditions	✓	✓	✓
Provide answers to medical questions	✓	✓	✓
Facilitate dialogue between patients to patients, and patients and health professionals		✓	✓
Collect data on patient experiences and opinions		✓	✓
Used for health intervention, health promotion and health education	✓	✓	✓
Reduce stigma		✓	✓
Provide online consultations		✓	✓

**Table 4:** Benefits of using social media for health communication for the general public, patients, and health professionals.

Benefits of social media for health communication	Social media user		
	General Public	Patients	Health Professionals
Increase interactions with others	✓	✓	✓
More available, shared, and tailored information	✓	✓	✓
Increase accessibility & widening access	✓	✓	✓
Peer/social/emotional support	✓	✓	✓
Public health surveillance	✓	✓	✓
Potential to influence health policy	✓	✓	✓

**Table 5:** Limitations of social media for health communication among the general public, patients, and health professionals.

Limitations of social media for health communication	Social media user		
	General Public	Patients	Health Professionals
Lack of reliability	✓	✓	✓
Quality concerns	✓	✓	✓
Lack of confidentiality & privacy	✓	✓	✓
Often unaware of the risks of disclosing personal information online	✓	✓	
Risks associated with communicating harmful or incorrect advice using social media	✓	✓	
Information overload	✓	✓	
Not sure how to correctly apply information found online to their personal health situation	✓	✓	
Certain social media technologies may be more effective in behavior change than others	✓		
Adverse health consequences	✓		
Negative health behaviors	✓		
Social media may act as a deterrent for patients from visiting health professionals		✓	✓
Currently may not often use social media to communicate to patients			✓

## Discussion

The 98 research studies in this review provided evidence that social media (most reported applications were Facebook, Blogs, Twitter, and YouTube) can create a space to share, comment, and discuss health information on a diverse range of health issues such as sexual health, diabetes, flu/H1N1, and mental health issues [24-121]. Social media attracts a large number of users thus creating a platform for mass health communication [35] with identified uses, benefits, and limitations for the general public, patients, and health professionals.

### Uses of Social Media for Health Communication

The main uses of social media focus on increasing interactions with others, and facilitating, sharing, and obtaining health messages [24-121]. The general public mainly use social media for themselves, family members, and/or friends to obtain and share information on a wide range of health issues [36,60,61,71,74,103]. Patients can share their experiences through discussion forums, chat rooms and instant messaging, or online consultation with a qualified clinician [26,62,63]. Some health professionals were reported to use social media to collect data on patients [26,65] and to communicate with patients using online consultations [88]; however, this latest use is limited. Recent research reported that female health professionals in Quebec, Canada, believed that Web 2.0 may be a useful mechanism for knowledge transfer but is limited due to their lack of time and technological skills [126]. Perhaps in light of Kaplan and Haenlein’s [10] classifications of social media, further work on improving the “social presence”, the closeness to synchronous face-to-face communication of such online consultations, would contribute to improving communication between health professionals and patients. Another recent study applied social network analysis to understand the knowledge sharing behavior of practitioners in a clinical online discussion forum and found that although their number is limited, interprofessional and interinstitutional ties are strong [127]. This relates to Gilbert and Karahalios’ [128] social tie analysis and suggests that development of mechanisms that evaluate tie strength in social media that in turn impact on its functionality may be useful for health communication. Further technological advances will provide more opportunities to use social media in health care in the future, especially between patients and patients, and also health professionals and patients. However, both patients and health professionals may require training to fully maximize the uses of using social media in health care.

### Benefits of Social Media for Health Communication

Numerous benefits of using social media for health communication were reported for the general public, patients, and health professionals. A major benefit of social media for health communication is the accessibility and widening access of health information to various population groups, regardless of age, education, race or ethnicity, and locality, compared to traditional communication methods [35,64,95,72]. While these changing patterns may lessen health disparities, traditional inequalities in overall Internet access remain. Furthermore, variation in social media engagement according to personality traits, age, and gender [15] suggests the need for ongoing scrutiny regarding equality of access and effectiveness for different users. Social media can be used to provide a valuable and useful source of peer, social, and emotional support to individuals, including those with various conditions/illnesses [48,62,71]. Hwang and colleagues [62] reported that encouragement, motivation, and shared experience were important social support features of social media sites.

Social media allows users to generate peer-to-peer discussion in a way not enabled by traditional websites [48,50,62,71]. However, this may challenge expectations, relationships, quality, and consistent health care practice. As Moen et al [44] explain, current patterns of collaboration tend to produce an asymmetric patient-health care provider relationship. This highlights a strong need for health providers to maintain a role within social media health communication that is not simply the same as that of patient and general public users. Keitzmann et al [17] have suggested that organizations need to recognize and understand the social media landscape, and where the conversations about them are already being held (cognize), develop strategies that are suitable, work out how often and when they should enter into conversations, and be aware of what others are doing and act accordingly. This review highlights clearly that social media has benefits for health communication but the long-term effects are not known. As the use of social media is expected to increase in the future [1], there may be further benefits of using social media in health care. It is not yet known how effective social media applications are in health communications, which warrants further research.

### Limitations of Social Media for Health Communication

Social media tools remain informal, unregulated mechanisms for information collection, sharing, and promotion, so the information is of varying quality and consistency [26,27,39,40,42,44,47,50,69,74,85,95]. Similar issues exist with traditional Internet sites, but these issues are being heightened by the interactive nature of social media, which allows lay-users to upload information regardless of quality [50]. Reliability may be monitored by responsible bodies using automated processes, employed to signal when content has been significantly edited, and progress is being made in automated quality detection [50]. Further work to improve the “media richness” [10] of social media for health communication, that is, how they may reduce ambiguity and uncertainty, would be valuable. In addition, combining more resources in one site could improve reliability of information. As patients interact and share links, they could compare numerous social media sites and triangulate information to help them discern correct from incorrect information [50]. Despite concerns, information found on some websites is reported to be generally factually accurate [39,62]. A further limitation is that postings can be a permanent record and be viewed by an increasing audience, and perhaps users are unaware of the potential size of the audience base. Regulatory and security issues must be addressed to broach a way forward for best-practice that allows the benefits of social media to be utilized yet still protects patients’ privacy and to therefore improve use of these media in routine clinical care. This is a public policy issue and is already being contested in the United States. Public education is required for the general public, patients, and health professionals to make them more aware of the nature of using social media. Consideration of the variation in social media engagement according to personality traits, age, and gender [15] will be valuable in tailoring education to meet the needs of population groups.

### Gaps in the Research Literature and Recommendations of Research Into Social Media for Health Communication

This literature review has shown that the general public, patients, and health professionals use social media in health care for various purposes with numerous benefits and limitations. The current research’s methodological scoring was low; this was mainly due to the fact that the vast majority of the studies in this review were exploratory and descriptive. To date, there is very limited evidence from RCTs and longitudinal studies. To more fully determine the role of social media for health communication, further research with larger sample sizes and more robust methodologies are required. Based on this review [24-121], several gaps in the literature have been identified that need to be addressed:

- the impact of social media for health communication in specific population groups, such as minority groups, patients groups, culture differences;
- the relative effectiveness of different applications of social media for health communication;
- the longer-term impact on the effectiveness of social media for health communication;
- the most suitable mechanisms to monitor and enhance the quality and reliability of health communication using social media;
- the risks arising from sharing information online, the consequences for confidentiality and privacy, and the most suitable mechanisms for effectively educating users in the maintenance of their confidentiality and privacy;
- the full potential of social media in effectively supporting the patient-health professional relationship;
- the impact of peer-to-peer support for the general public, patients, and health professionals to enhance their interpersonal communication;
- the impact of social media on behavior change for healthy lifestyles.

To address these gaps in the literature, the key recommendations for future health communication research focus on robust and comprehensive evaluation and review, using a range of methodologies. The research priorities are highlighted below:

- To determine the impact of social media for health communication in specific population groups with large sample sizes (representation of population groups).
- To determine the relative effectiveness of different social media applications for health communication using RCTs.
- To determine the longer-term impact on the effectiveness of social media for health communication using longitudinal studies.
- To explore potential mechanisms for monitoring and enhancing the quality and reliability of health communication using social media.
- To investigate the risks arising from sharing information online and the consequences for confidentiality and privacy, coupled with developing the most suitable mechanisms to effectively educate users in the maintenance of their confidentiality and privacy.
- To determine how social media can be effectively used to support the patient-health professional relationship.
- To determine the impact of peer-to-peer support for the general public, patients, and health professionals to enhance their interpersonal communication.
- To explore the potential for social media to lead to behavior change for healthy lifestyles to inform health communication practice.

### Conclusions

Social media brings a new dimension to health care, offering a platform used by the public, patients, and health professionals to communicate about health issues with the possibility of potentially improving health outcomes. Although there are benefits to using social media for health communication, the information needs to be monitored for quality and reliability, and the users’ confidentiality and privacy need to be maintained. Social media is a powerful tool that offers collaboration between users and a social interaction mechanism for a range of individuals. With increasing use of social media, there will be further opportunities in health care. Research into the application of social media for health communication purposes is an expanding area because increasing general use of social media necessitates that health communication researchers match the pace of development. Further robust research is required to establish whether social media improves health communication practices in both the short and long terms.

## Multimedia Appendix 1

Excluded studies.

## Multimedia Appendix 2

Summary of the selected studies (n=98).

## Multimedia Appendix 3

Study quality scores using Downs and Black scale: checklist for measuring study quality (n=50).

## Abbreviations

PRISMA: Preferred Reporting Items for Systematic Reviews and Meta-Analyses
RCT: randomized controlled trial
