# Supplementary material for: A New Dimension of Health Care: Systematic Review of the Uses, Benefits, and Limitations of Social Media for Health Communication
Source: J Med Internet Res. 2013 Apr 23;15(4):e85. doi: 10.2196/jmir.1933 (PMC3636326; doi:10.2196/jmir.1933)
Supplement: Supplementary file 1 [file jmir_v15i4e85_app1.pdf]

Multimedia Appendix 1. Excluded studies

A new dimension of healthcare: Review of the uses, benefits and limitations of social media for Health Communication

S. Anne Moorhead PhD; Diane E Hazlett PhD; Laura Harrison MSc; Jennifer K Carroll MD, MPH; Ciska Hoving PhD

EXCLUDED ARTICLES (n=50)

SUMMARY OF REASONS (n=13):

- Teaching focus (10)
- Not / not primarily health communication (8)
- Marketing / advertising focus (5)
- Social networks not social network websites (5)
- Article (i.e. not primary research study) (5)
- Research focus e.g. on participant recruitment (4)
- Review paper (3)
- Not focused on social media (3)
- Not communication focused (2)
- Technology focused (2)
- Internet-based / web 1.0 (1)
- Study protocol (1)
- Personal use, not for communication purposes (1)

TEACHING FOCUS (n=10)

1. Creutzfeldt J, Hedman L, Medin C, Heinrichs WL, Fellander-Tsai L. Exploring virtual worlds for scenario-based repeated team training of cardiopulmonary resuscitation in medical students. J Med Internet Res 2010;12(3):e38. doi:10.2196/jmir.1426.
2. Gray K, Annabell L, Kennedy G. Medical students' use of Facebook to support learning: insights from four case studies. Med Teach 2010;3(12):971-6. Accession Number - 21090950.
3. Sandars JJ, Schroter SS. Web 2.0 technologies for undergraduate and postgraduate medical education: an online survey. Postgrad Med J 2007;83(986):759-62. Accession number – 18057175.
4. George DR, Dellasega C. Social media in medical education: two innovative pilot studies. Med Educ 2011:45(11):1158-9. doi: 10.1111/j.1365-2923.2011.04124.x.
5. Estus EL. Using Facebook within a geriatric pharmacotherapy course. Am J Pharm Educ 2010;74(8):145. PMCID: PMC2987285.
6. Fox BI, Varadarajan R. Use of twitter to encourage interaction in a multi-campus pharmacy management course. Am J Pharm Educ 2011;75(5):88. doi: 10.5688/ajpe75588.
7. Gray K, Tobin J. Introducing an online community into a clinical education setting: a pilot study of student and staff engagement and outcomes using blended learning. BMC Med Educ 2010;10:6. doi: 10.1186/1472-6920-10-6.
8. Haines SL, Van Amburgh JA. A video casting project to promote the pharmacist's role in public health. Am J Pharm Educ 2010 74(6):97. PMCID: PMC2933027.

9. Varga-Atkins T; Dangerfield P, Brigden D. Developing professionalism through the use of wikis: A study with first-year undergraduate medical students. Med Teach 2010;32(10):824-9. doi:10.3109/01421591003686245
10. Cinnamon J, Schuurman N. Injury surveillance in low-resource settings using geospatial and social web technologies. Int J Health Geographics 2010;9:25. doi:10.1186/1476-072X-9-25.

NOT / NOT PRIMARILY HEALTH COMMUNICATION (n=8)

1. Konovalov S, Scotch M, Post L, Brandt C. Biomedical informatics techniques for processing and analyzing web blogs of military service members. J Med Internet Res 2010;12(4):e45. doi:10.2196/jmir.1538.
2. Moreno MA, Jelenchick LA, Egan KG, Cox E, Young H, Gannon KE, Becker T. Feeling bad on Facebook: depression disclosures by college students on a social networking site. Depression Anxiety 2011; 28(6):447-55. doi: 10.1002/da.20805.
3. Sublet VV, Spring CC, Howard JJ. Does social media improve communication? Evaluating the NIOSH science blog. Am J Indust Med 2011;54(5):384-94. doi: 10.1002/ajim.20921.
4. Thompson LA, Black E, Duff WP, Black NP, Saliba H, Dawson K. Protected health information on social networking sites: ethical and legal considerations. J Med Internet Res 2011;13(1)e8. Accession number- 201111253.
5. Webster YW, Dow ER, Koehler J, Gudivada RC, Palakal MJ. Leveraging health social networking communities in translational research. J Biomed Inform 2011;44 (4):536-44. doi: 10.1016/j.jbi.2011.01.010.
6. Forsyth SR, Malone RE. “I'll be your cigarette—Light me up and get on with it”: Examining smoking imagery on YouTube. Nicotine Tobacco Res 2010;12(8) 810–6. doi: 10.1093/ntr/ntq101.
7. Thompson LA, Dawson K, Ferdig R, Black EW, Boyer J, Coutts J, Black NP. The intersection of online social networking with medical professionalism. J Gen Intern Med 2008;23(7):954-7. PMID: 18612723.
8. Huang C, Chan E, Hyder AA. Web 2.0 and Internet social networking: a new tool for disaster management? - Lessons from Taiwan. BMC Med Inform Decis Making 2010;10(1):57. Accession number- 20925944.

MARKETING / ADVERTISING FOCUS (not health communication-focused; n=5)

1. Gosselin P, Poitras P. Use of an Internet “viral” marketing software platform in health promotion. J Med Internet Res 2008;10(4):e47. doi:10.2196/jmir.1127.
2. Patel A, Seo J, Yang M, Sansgiry S. Content analysis of digital videos featuring prescription drug advertisements on Internet social media-YouTube. J Am Pharm Assoc 2010; 50(2):244-5. doi: 10.1331/JAPhA.2010.10511.
3. Freeman B, Chapman S. Open source marketing: camel cigarette brand marketing in the "Web 2.0" world. Tobacco Control 2009; 18(3): 212-217. doi:10.1136/tc.2008.027375.
4. Purdy CH. Using the internet and social media to promote condom use in Turkey. Reprod Health Matters 2011;19(37):157-165. PII: S0968-8080(11)37549.
5. Liang BA, Mackey TK. Prevalence and global health implications of social media in direct-to-consumer drug advertising. J Med Internet Res 2011;13(3):e64. doi: 10.2196/jmir.1775.

SOCIAL NETWORKS NOT SOCIAL NETWORK WEBSITES (n=5)

1. Rice E, Milburn NG, Monro W. Social networking technology, social network composition, and reductions in substance use among homeless adolescents. Prev Sci 2011;12(1):80-88. doi: http://dx.doi.org/10.1007/s11121-010-0191-4.
2. Cauchemez S, Bhattarai A, Marchbanks TL, Fagan RP, Ostroff S, Ferguson NM, Swerdlow D, Sodha SV, Moll ME, Angulo FJ, Palekar R, Archer WR, Finelli L. Role of social networks in shaping disease transmission during a community outbreak of 2009 H1N1 pandemic influenza. Natl Acad Sci USA 2011;108(7):2825-30. Doi: http://dx.doi.org/10.1073/pnas.1008895108.
3. Christakis NA, Fowler JH. Social network sensors for early detection of contagious outbreaks. PLoS One 2010;5(9):e12948. doi: 10.1371/journal.pone.0012948.
4. Valente TW, Fujimoto K, Chou CP, Spruijt-Metz D. Adolescent affiliations and adiposity: a social network analysis of friendships and obesity. J Adolescent Health 2009;45(2):202-4. PMCID: PMC2747768.
5. Zheng K, Pacman R, Krackhardt D, Johnson MP, Diamond HS. Social networks and physician adoption of electronic health record: insights from an empirical study. J Am Inform Assoc 2010;17:38-336. doi: 10.1136/jamia.2009.000877.

ARTICLE (i.e. not primary research study) (n=5)

1. Bastida R, McGrath I, Maude P. Wiki use in mental health practice: recognizing potential use of collaborative technology. Int J Ment Health Nurs 2009;19(2):142-8. doi: 10.1111/j.1447-0349.2009.00655.x.
2. Young SD. Recommendations for using online social networking technologies to reduce inaccurate online health information. Online J Health Allied Sci 2011;10(2):2. PMCID: PMC3196338.
3. Boulos MNK, Scotch M, Cheung KH, Burden D. Web GIS in practice VI: a demo playlist of geo-mashups for public health neogeographers. Int J Health Geography 2008;7:38. doi: 10.1186/1476-072X-7-38.
4. Angelle D. Conversations with the community: the Methodist hospital system's experience with social media. Front Health Serv Manage 2011;28(2):15-21. PMID: 22256507.
5. Sharp J. Brand awareness and engagement: a case study in healthcare social media. Front Health Serv Manage 2011;28(2):29-33. Accession number – 22256509.

RESEARCH FOCUS (e.g. on participant recruitment; n=4)

1. Gunasekaran B, Jayasinghe Y, Moore E, Fenner Y, Fletcher A, Tabrizi S, Wark JD, Garland SM. Knowledge of human papillomavirus (HPV) and the HPV vaccine among young women in victoria, australia who have been randomly recruited via facebook. J Pediatr Adolescent Gynecol 2011;24(2):e66-7. doi: http://dx.doi.org/10.1016/j.jpag.2011.01.
2. Resnick PJ, Janney AW, Buis LR, Richardson CR. Adding an online community to an Internet-mediated walking program. Part 2: strategies for encouraging community participation. J Med Internet Res 2010;12(4):e72. doi: 10.2196/jmir.1339.
3. Levine D, Madsen A, Wright E, Barar RE, Santelli J, Bull S. Formative research on MySpace: online methods to engage hard-to-reach populations. J Health Commun 2011;16(4):448-54. doi: 10.1080/10810730.2010.546486.
4. Tweet MS, Gulati R, Lee A, Hayes SN. Spontaneous coronary artery dissection: a disease-specific, social networking community-initiated study. Mayo Clinic P 2011;86(9):845-50. Accession number- 21878595.

REVIEW PAPER (n=3)

1. Gold JJ, Pedrana AE, Sacks-Davis RR, Hellard ME, Chang SS, Howard S, Keogh L, Hocking JS, Stoove MA. A systematic examination of the use of online social networking sites for sexual health promotion. BMC Public Health 2011;11:583. Accession number – 21777470.
2. Fernandez-Luque LL, Karlsen RR, Bonander JJ. Review of extracting information from the social web for health personalization. J Med Internet Res 2011;13(1):e15. doi: 10.2196/jmir.1432.
3. Hughes B, Joshi I, Wareham J. Health 2.0 and Medicine 2.0: tensions and controversies in the field. J Med Internet Res 2008;10(3). doi: doi:10.2196/jmir.1056.

NOT FOCUSED ON SOCIAL MEDIA (n=3)

1. Wilson SL, Huttlinger KK. Pandemic flu knowledge among dormitory housed university students: a need for informal social support and social networking strategies. Rural Remote Health 2010;10(4):1526. Accession number – 21028934.
2. Wicks P, Massagli M, Kulkarni A, Dastani H. Use of an online community to develop patient-reported outcome instruments: the multiple sclerosis treatment adherence questionnaire (MS-TAQ). J Med Internet Res 2011;13(1):e12. doi:10.2196/jmir.1687.
3. Fukuoka Y, Kamitani E, Bonnet K, Lindgren T. Real-time social support through a mobile virtual community to improve healthy behavior in overweight and sedentary adults: a focus group analysis. J Med Internet Res 2011;13(3):e49. doi:10.2196/jmir.1770

NOT COMMUNICATION FOCUSED (n=2)

1. Su, C. Can Social networking improve individual competitiveness? exploring the effects of social network centralities on knowledge acquisition and work efficiency in organizational work teams. Competition Forum 2011;9(2):247-54. ProQuest document ID – 912867886.
2. Centola D. The spread of behavior in an online social network experiment. Science 2010;29:1194-7. doi: 10.1126/science.1185231. (TECHINICAL).

TECHNOLOGY FOCUSED (n=2)

1. Doing-Harris KM, Zeng-Treitler Q. Computer assisted update of a consumer health vocabulary through mining of social network data. J Med Internet Res 2011;3(2):e37. doi:10.2196/jmir.1636.
2. Cheung KH, Yip KY, Townsend JP, Scotch M. HCLS 2.0/3.0: Health care and life sciences data mashup using Web 2.0/3.0. Comput Biomed Res 2008;41(5):694-705. doi: 10.1016/j.jbi.2008.04.001.

INTERNET BASED / WEB 1.0 (n=1)

1. Wen KY, Kreps G, Zhu F, Miller S. Consumers' perceptions about and use of the Internet for personal health records and health information exchange: analysis of the 2007 Health Information National Trends Survey. J Med Internet Res 2010;12(4):e73. doi: 10.2196/jmir.1668.

STUDY PROTOCOL (n=1)

1. Killackey E, Anda AL, Gibbs M, Alvarez-Jimenez M, Thompson A, Sun P, Baksheev GN. Using Internet enabled mobile devices and social networking technologies to promote exercise as an intervention for young first episode psychosis patients. BMC Psychiatry. 2011;11:80. Doi: http://dx.doi.org/10.1186/1471-244X-11-80.

PERSONAL USE, NOT FOR COMMUNICATION PURPOSES (n=1)

1. MacDonald J, Sohn S, Ellis P. Privacy, professionalism and Facebook: a dilemma for young doctors. Med Educ 2010;44(8):805-13. PMID: 20633220.
