# Supplementary material for: A New Dimension of Health Care: Systematic Review of the Uses, Benefits, and Limitations of Social Media for Health Communication
Source: J Med Internet Res. 2013 Apr 23;15(4):e85. doi: 10.2196/jmir.1933 (PMC3636326; doi:10.2196/jmir.1933)
Supplement: Supplementary file 2 [file jmir_v15i4e85_app2.pdf]

Multimedia Appendix 3. Summary of the selected studies (n=98)

Study	Study design	Social media tool /application	Study purpose	Participants / sample & sample size	Measurement tools	Results	Conclusion	Use of social media[1]
Freeman & Chapman (2007) [14]	Descriptive (qualitative content analysis)	YouTube	To analyze the content of smoking imagery on YouTube	50 YouTube videos with smoking content	Content Analysis of YouTube videos	52% videos contained tobacco smoking imagery<br><br>Viewer feedback expressed a wide range of opinions about smoking; most of which were positive (pro-smoking)	Smoking imagery is abundant on YouTube	YouTube videos contain a range of information about smoking, including messages that smoking
Moreno et al. (2007) [15]	Cross-sectional observational study (qualitative)	MySpace	To examine publicly available 16 & 17 year old MySpace web profiles & determine the prevalence of personal risk behavior descriptions / identifiable information	142 publicly available MySpace web profiles	Content analysis of MySpace web profiles	47% of profiles contained risk behavior information<br>97.2% contained personally identifying information	Majority of 16 & 17 year old MySpace profiles contain identifiable information; half include risk behavior information	MySpace has potential use for health interventions targeting at-risk teens
Adams (2008) [16]	Case Study (qualitative)	Blogs	To explore the diverse purposes for which blogging applications can be (or are being) used in relation to health & introduce the idea of "health goal-oriented" blogging	2 health educators & 1 project leader<br><br>2 blog sites	Semi-structured interviews & content analysis of two blog sites	Quality of information / exposure of patients to incorrect health information is a primary concern<br><br>Most information posted on blogs was provided by lay users	Blogging provides opportunities for health communication practice & health organizations	Blog sites create a space where individuals can access tailored resources to deal with health issues, & offer their own information for use by others<br><br>Organizations can aggregate data about patient experiences from blogs
Clauson et al. (2008) [17]	Mixed methods	Wikipedia	To compare the scope,	Wikipedia site & MDR	Questionnaire	Wikipedia was able to answer significantly fewer drug information	Wikipedia has narrower scope, is less complete & has more errors of omission	Wikipedia may be a useful point of engagement for

	(quantitative & qualitative)		completeness & accuracy of drug information in Wikipedia with that of a free online, traditionally edited database, Medscape Drug Reference (MDR)	database	Wikipedia site<br><br>MDR database	questions (40%) compared with MDR (82.5%)	than MDR but is not authoritative & should only be used as a supplementary source of drug information	consumers
Frost et al. (2008) [18]	Descriptive (qualitative content analysis)	Patientslikeme	To describe the components & design of the online community<br><br>Identify & analyze how users of this platform reference personal health information within patient-to-patient dialogues	123 comments from members on Patientslikeme	Content analysis of comments from members on Patientslikeme	Members used data to locate others with specific health experiences to: answer questions; offer personally acquired disease management knowledge to those most likely to benefit; build & maintain relationships based on shared concerns	Can help patients engage in dialogue to inform disease self-management<br><br>Recommended that future designs automate matching of patients with similar conditions / treatments & integrate data into online platforms for health conversations	Patientslikeme enables patients to engage in dialogue with other patients & share health information & advice
Kovic et al. (2008) [19]	Survey (quantitative)	Medical blogs	To contribute to a better understanding of the medical blogosphere by investigating the characteristics of medical bloggers & their blogs	197 medical bloggers of English-language medical blogs	Survey	Majority of bloggers were white (75%); highly educated (71% with a Master's degree or doctorate); male (59%); residents of the United States (72%); between the ages of 30-49 (58%); working in the healthcare industry (67%) & experienced bloggers (23% blogging for 4 years or more; 38% for 2 or 3 years; 32% for about 1 year; only 7% for 6 months or less)	Medical bloggers are highly educated & committed blog writers	Medical blogs are frequently noticed by mainstream media & may influence medical & health policy
Lagu et al. (2008) [20]	Descriptive (qualitative content analysis)	Medical blogs	To examine the scope & content of medical blogs & approximate how often blog authors commented about patients, violated patient privacy, or displayed a lack of professionalism	5 entries from 271 medical blogs, apparently written by physicians or nurses	Content analysis of medical blogs, apparently written by physicians or nurses	42.1% blogs described individual patients<br><br>16.6% blogs described encounters with individual patients included enough information for patients to identify their doctors or themselves	Health professional's risk revealing too much information, in their tone or content, & risk reflecting poorly on blog authors, & on their profession	Blogs provide physicians & nurses with an opportunity to share their narrative

Scotch et al. (2008) [21]	Case Report (qualitative)	Web 2.0 application	To assess the feasibility of using Web 2.0 technologies to develop complex public health applications	Publicly available data from 3websites (used to create Web 2.0 application)	Report of application development process	Application often failed due to varied timeouts for application response across web sites & services, internal caching errors, & software added to web sites by administrators to manage the load on their servers	Web 2.0 applications are not mature enough for large-scale public health data applications	Web 2.0 / grid computing approaches in health informatics has potential value
Tan (2008) [22]	Qualitative	Blog posts	How one might blog within a social network as a form of self-therapy?	A blogger	Interview	Blogging is interactive & offer a promise of listening, empathy, understanding & belonging to a wider community	Blogging could potentially be used as a form of self- help therapy	Blogging has potential for self- help therapy as an opportunity for self-reflection & sympathetic relief through expressing emotions
Timpka et al. (2008) [23]	Participatory action research (mixed methods)	Web 2.0 system	Address the potential to develop Web 2.0 services for young persons with a chronic disease	Researchers from medical informatics, pediatrics, & behavioral science (number not stated) Clinicians (number not stated) Patients (number not stated)	Interviews<br><br>Surveys<br><br>Cultural probe & card sorting methods	Design elements enable access to materials for development of the competences needed for chronic disease management in specific community setting	A model based architecture for Web 2.0 systems for chronic disease management was built & described	Web 2.0 has potential use for chronic disease management
Beard et al. (2009) [24]	Descriptive (qualitative content analysis)	Second Life	To survey & categorize the range of health-related activities on Second Life; to examine the design attributes of the most innovative & popular sites; & to assess the potential utility of Second Life for the dissemination of health information &	68 health related sites on Second Life	Content analysis of health related sites on Second Life	A wide range of health-related activities & diverse group of users, including organizations, groups & individuals were identified<br><br>Main type of site's principle aim was patient education, or to increase awareness about health issues, followed by support sites, training & marketing sites	Users are engaged in a wide range of health activities on Second Life, which may be impacting health behaviors	Second Life can be used for health communication, including patients education, to increase awareness of health issues, & social support

			health behavior change					
Chou et al. (2009) [25]	Secondary data analysis using Health Information National Trends Survey (HINTS) 2007 (quantitative)	Online support groups; Blogs; Social networking sites	To identify the socio demographic & health related factors associated with current adult social media users in the Unites States	7674 members of the general public	Secondary data analysis using HINTS 2007 using telephone interviews	5% US adults reported participating in an online support group, 23% reported using a social networking site & 7% reported blogging<br><br>Social media use was most prevalent among the 18-24 years age group<br><br>Support group participation was predicted by age, poorer subjective health, & a personal cancer experience	Social media are accessing the US population, regardless of education, race/ethnicity, or health care access & may be changing the communication pattern in the US	Social media may be used to access minority ethnic / racial groups, & young people to communicate health messages
Denecke & Nejdl (2009) [26]	Descriptive (qualitative content analysis)	Medical question & answer portals, Weblog , Medical reviews & Wikis	To provide an overview of medical content available on the web	9600 questions from medical question & answer portals<br><br>95 weblogs (5274 patients written posts, 7852 physician written posts, 5724 nurse written posts)<br><br>3731 drug medical reviews for 630 drugs<br><br>725 pages of AskDrWiki& 750 pages from Medline Plus	Content analysis of questions from medical question & answer portals	Weblogs & answer portals deal mainly with diseases & medications whereas the Wiki & encyclopedia provide more information on anatomy & procedures<br><br>Patients & Nurses describe personal aspects of their lives<br><br>Doctors aim to present health-related information in their blog posts	Knowledge on information content / content differences can be used by search engines to improve ranking, & direct users to relevant information resources	Web contains health information which can provide answers to medical questions
Farmer et al. (2009) [27]	Descriptive (qualitative content analysis)	Facebook	To ascertain whether Facebook had user groups that are connected to common medical conditions, & to classify the user	290962 individual Facebook users who were part of 757 groups	Content analysis of Facebook groups	Patient groups accounted for 47.4% , patient/ carer support groups 28.1%, fundraising groups 18.6% & others 5.8%	Facebook is readily accessible portal for patients, carers, & healthcare professionals to share their experiences of exploration, diagnosis & management of disease	Facebook is being used by patients, carers & health professionals to share their experiences of disease management, exploration & diagnosis<br><br>Facebook is also being used

			groups that were identified as well as enumerating the number of users contained therein					for research, education & fundraising
Fernandez-Luque et al. (2009) [28]	Descriptive (qualitative content analysis)	YouTube	To evaluate the characteristics of patient generated videos found within YouTube video comments	25 Multiple Sclerosis patient generated videos	Content analysis of Multiple Sclerosis patient generated videos	Public use social media to share personal health information online, commenting on medications, symptoms & diagnoses	Concerns are raised about the quality of information posted on YouTube by lay users	YouTube videos are used by the public to share health information on medications, symptoms & diagnoses
Hughes et al. (2009) [29]	Mixed methods	Web 2.0	To examine Web 2.0 use by 35 junior physicians in clinical settings to further understand their impact on medical practice	35 junior physicians	Survey<br>Semi-structured diaries<br>Semi-structured interviews	53% of internet visits employed Web 2.0 content, which has a role in information seeking for both clinical decisions & medical education	Potential risks of poor quality health information using Web 2.0 & its effectiveness depends on how it is used by physicians	Web 2.0 is used by junior physicians to seek health information to inform clinical decisions & medical education
Jennings et al. (2009) [30]	Pilot intervention (mixed methods)	SNS (discussion boards & synchronous chat)	To assess the feasibility, acceptability, & effectiveness of an internet-based virtual clinic designed to facilitate self-management in patients who used insulin pumps to manage their diabetes	17 patients with diabetes	Pre- and post-questionnaires (n=17 participants)<br>Interviews (n=5)	Participants found the virtual clinic easy to use<br>Peer support was the most valued aspect & discussion boards the most used component	Internet-based system to aid management of diabetes appears feasible & well accepted by patients	Online discussions along diabetic patients aid in the management of diabetes
Keelan et al. (2009) [31]	Descriptive (qualitative content analysis)	Blogs	To better understand the public's reaction to the Human Papiloma Virus vaccine	303 blogs	Content analysis of blogs	52% of bloggers were classified as positive, 43% as negative, & 17% as ambivalent towards immunization	Blog analysis is helpful for public health officials to monitor public opinion, & design health education to combat negative views, & has potential in improving communication	Public health officials can use blogs to monitor public reaction about health issues
Kim (2009) [32]	Descriptive (qualitative content analysis)	Cancer blog posts	To explore the efficacy of user-defined subject tagging &	485 cancer blog posts	Content analysis of cancer blog posts	Both blogger-defined subject tags & software-generated subject tags were often too broad or too narrow in focus, to produce effective search	Resources such as blogs are different than mainstream communication channels in terms of topics & subject access methods & therefore different	Blogs can be used for health communication

			software generated subject tagging for describing & organizing cancer blog contents			results for those seeking to extract cancer information from blog posts	search methods need to be employed (by medical librarians) to identify relevant tags & needs to be more easily accessed	
Lupianez-Villanueva et al. (2009) [33]	Mixed methods	Web 2.0 initiatives	To explore the opportunities & challenges of Web 2.0 within the health care system & to identify the gap between the potential of these online activities & applications & the empirical data	4,319 physicians, nurses, pharmacists, patient support groups completed the survey<br><br>Analysis of 1045 pages	Online survey<br>Static web shot analysis & exploration of Web 2.0 initiatives	Gap identified in the use of Web 2.0, primarily the lack of interactions in health care communications	Web 2.0 has potential to improve communication within health care settings	Web 2.0 can be used within healthcare settings & may potentially improve communication
Moen et al. (2009) [34]	Exploratory participatory design (Qualitative)	Web 2.0	To create a collaborative environment for peer support & knowledge construction relating to a condition	50 participants	Workshop interviews (internal development blog & discussions about mock ups)	Knowledge, suggestions & practical experiences seen as being equal in value, challenging the asymmetric patient-healthcare provider relationship<br><br>Concerns about accountability, confidentiality & trust were highlighted	Web 2.0 provides opportunities to advance peer support<br><br>Qualities of relationships & collaboration in healthcare need to be considered in implementation	Web 2.0 can be leveraged for peer support about health matters
Moreno et al. (2009a) [35]	RCT (quantitative)	MySpace	To determine whether an online intervention reduces references to sex & substance abuse on social networking web sites among at-risk adolescents	190 18-20 year olds with public MySpace profiles who met criteria for being at risk<br><br>2 groups:<br>1.Mail intervention (intervention was a single physician email)<br>2.Control (no intervention)	Web profiles evaluated for references to sex & substance misuse before & after intervention	References to sex & substance use ceased in 13.7% of profiles in the intervention group vs. 5.3% in the control group for sex & 26% vs. 22% for substance abuse	Brief email intervention showed promise in reducing sexual references in the online profile of at-risk adolescents	MySpace profiles contain sex & alcohol abuse references by adolescents

Moreno et al. (2009b) [36]	Cross sectional study (qualitative)	MySpace	To determine the prevalence of & associations among displayed risk behavior information that suggests sexual behavior, substance use & violence in a random sample of self-reported 18 year old adolescent publicly accessed MySpace profiles	500 publicly available web profiles of self-reported 18 year olds in the United States	Content analysis of publicly available web profiles	54% of profiles contained risk behavior information - 24% referenced sexual behaviors; 41% substance use; 14.4% violence	Adolescents on public websites frequently display risk behavior information	Adolescents display health risk behavior information on MySpace<br><br>Social networking Sites may have use in health promotion
Nordqvist et al. (2009) [37]	Qualitative	Web 2.0 portal	To explore pediatric practitioner's attitudes towards the introduction of a local Web portal for providing young type 1 diabetes patients with interactive pedagogic devices, social networking tools, & locally produced self-care & treatment information; including opportunities & barriers	20 clinicians (7 doctors, 9 nurses, 2 dieticians, 2 welfare officers) from 2 pediatric diabetes teams	Semi-structured telephone interviews	Online peer-to-peer interactive communication was seen as a valuable function<br><br>Clinicians did not expect the portal would be used extensively for social networking amongst patients	Web 2.0 portal provided information, & practical & social support<br><br>Collaboration is encouraged between all user groups<br><br>Training in Web 2.0 in healthcare is required for clinicians	Web 2.0 portal can be used to provide patients with health information & to peer-to-peer facilitate practical & social support
Takahashi et al. (2009) [38]	Mixed methods	SNS	To explore the potential benefits & harms of a SNS for depression	37 SNS users	Questionnaire Qualitative content analysis,<br>Social network analysis	Peer support could be passive, active or interactive<br><br>A downward depressive spiral was related to friends who were moderately or severely depressed & friends with negative assessment of the SNS	A SNS for people with depression can provide support that meets user's needs	SNS can provide peer support for those suffering form depressive illnesses

Versteeg et al. (2009) [39]	Observational (Qualitative)	MySpace	To describe the content of asthma-related interest groups on MySpace	188 publicly available MySpace asthma groups	188 publicly available MySpace asthma groups	36 (19%) directly related to asthma<br><br>Only one main group contained reference clinical information<br><br>42% shared a personal story, about diagnoses, triggers, problems & experiences	Asthma interest groups contained questions, advice & links to commercial web sites<br><br>Concerns - substantial misinformation	MySpace is being used to share health information, in particular personal stories & experiences
Adams (2010) [40]	Descriptive (qualitative content analysis)	Blogs & Wiki	To explore the diverse purposes for which blogging applications can be (or are being) used in relation to health	63 postings from 6 blogs & 1 wiki	Content analysis of postings from blogs & wiki	Issues for concern - Disclosure of authorship, information quality, anonymity & privacy, individual's ability to apply health information to their personal circumstances<br><br>Web 2.0 has advantages - the use of symbolic information for patient education, & to reach a range of patients	Web 2.0 provides opportunities for health communication by facilitating the creation & sharing of information by lay users<br><br>Concerns about reliability must be considered when incorporating these tools into health communication	Web 2.0 facilitates the creation & sharing of health information by lay users
Ahmed et al. (2010) [41]	Descriptive (qualitative content analysis)	Facebook	To scrutinize postings on Facebook groups related to concussion & examine the purpose of these postings	145 postings from 17 Facebook groups, related to concussion	Content analysis of Facebook group postings	Individuals mainly used Facebook to relate personal experiences of concussion (65%) & used to seek (8%) or offer (5%) advice	Rich information available of sites such as Facebook<br><br>Peer interaction may be the key of this health education medium	Facebook is used to share health information; peer interaction is the key utility of this medium
Avery et al. (2010) [42]	Survey (quantitative)	Social media tools/applications, e.g. social networking sites, blogs, discussion boards	To examine the extent to which social media are adopted within public health agencies, & moderators of adoption	281 public relations practitioners in public health departments serving 4 distinct sizes of communities – urban, suburban, large town, rural	Survey	Low adoption of social media technologies (17%) - urban communities exhibited highest adoption rates & lack of home Internet cited as main barrier	SNS are not widely used by public health PR practitioners	SNS have limited potential for communicating health information at community level as many people do not have home Internet access
Clauson et al. (2010) [43]	Descriptive (qualitative content analysis)	Blogs	To examine the characteristics of pharmacist blogs	44 pharmacists' blogs (5 most recent posts)	Content analysis of blog posts	No blogs contained patient-identifying information<br><br>Pharmacists most commonly used positive language to describe the	Majority of pharmacist's blogs contained some type of discussion of pharmacologic therapies	Pharmacists blogs contain information on pharmacologic therapies & describe the profession, other healthcare

						profession, other healthcare professionals & patients<br><br>Almost half blogs contained explicit or unprofessional language	Although pharmacists most commonly used positive language to describe the profession, other healthcare professionals & patients, the highest rates of critical language was about patients & other healthcare professionals	professionals & patients
Chew & Eysenbach (2010) [44]	Descriptive (quantitative content analysis)	Twitter	To monitor the use of the term 'H1N1'; conduct a content analysis of Tweets; & validate Twitter for real-time content, sentiment & public attention tracking tool	5,395 tweets, randomly selected 9 days, 4 weeks apart	Content analysis of 5,395 tweets	Posts about resources were most frequent (52.6%)<br><br>Several Twitter activity peaks corresponded with major news stories<br><br>Study results correlated well with H1N1 incidence data	Twitter has potential to conduct infodemiology studies for public health<br><br>2009 H1N1 tweets were mainly used to disseminate information & were also a source of opinions & experiences	Twitter can be used for real-time content analysis to monitor public opinion on health issues, & enable health authorities to respond to public concerns
Cobb et al. (2010) [45]	Network analysis methods (quantitative)	Quitnet	To evaluate the social network structure of Quitnet, & compared its characteristics to other known social networks	7569 Quitnet members who were active during a 60 day period, & their ties	7569 Quitnet members	Among 7569 participants, 103,592 connections to other members were identified<br><br>Social network integration was linked to being female, being older, having been in the system longer & being a non-smoker	Quitnet is a large SNS with the characteristics required for social support / influence to promote smoking cessation & abstinence<br><br>Influential sub-groups identified may provide targets for future network level health interventions	Quitnet facilitates social support & influence to aid smoking cessation & abstinence
Colineau & Paris (2010) [46]	Survey (quantitative)	Health-related social networking sites	To understand how & why people use health-related social networking sites	70 English-speaking participants (33 patients & 37 caregivers)	Online survey	Most people were seeking information about their medical condition online, interacting with health professionals to discuss sensitive information & complex issues, & seeking peer support (outside family / friends)<br><br>People who generally use SNS do not necessarily use health-related sites & vice-versa	Patients are now organizing themselves in groups within SNS, & sharing observations & helping each other<br><br>There is little evidence of the effectiveness of these communities on people's health	SNS create a participatory place for patients to interact with peers & health professionals about health matters & gain information & support
Corley et al. (2010) [47]	Links analysis & text mining (qualitative)	Web & Social media (WSM)	To monitor influenza-like illness in social media & to identify possible web & social	158,497,700 WSM items	Links analysis & text mining of WSM items	Text mining reveals trends in flu posts that correlate to real-world influenza-like illness patient report data	Text & structural data mining of WSM provides a novel disease surveillance resource	WSM can be used as a public health surveillance tool & for dissemination of pertinent health information

			media communities to participate in a public health response					to targeted communities
Ding & Zhang (2010) [48]	Comparative & descriptive (qualitative content analysis)	Social media content form US & Chinese government websites	To analyze the different ways government institutions, commercial internet portals & communities employed social media to communicate risks about the H1N1 flu at the beginning of the epidemic	Social media content form US & Chinese government websites & Chinese outbreak in mainstream newspapers & social media networks	Content analysis of social media & newspaper posting	U.S. CDC & DHSS used social media to interact with public during H1N1 flu epidemic & communicate key messages, enhancing outreach<br><br>Most frequently used tools were Facebook (41.4%) & Twitter (14.4%)	Social media can be used to strategically communicate one-way or limited two-way messages as part of risk communication plans	Social media can be used to strategically communicate one-way or limited two-way messages as part of risk communication plans & to disseminate personalized messages, immediately & accurately making outreach more effective
Ekberg et al. (2010) [49]	Case study (qualitative)	Web 2.0 system	To examine guidelines for the design of Web 2.0 systems for supporting diabetic adolescent's every day learning needs, in light of theory about information behavior & social learning	Diabetic adolescents (number not stated)	Design group discussions<br>Focus groups	Web 2.0 design was developed to support a community of practice & social learning	Informal learning has more significance for knowledge accumulation among adults than formal education or training	Web 2.0 offers a space for informal learning / health education to take place
Greene et al. (2010) [50]	Descriptive (qualitative content analysis)	Facebook	To evaluate the content of communication in Facebook communities dedicated to diabetes	15 most recent wall posts & 15 most recent discussion topics from the 10 largest Facebook groups focused on diabetes management	Content analysis of wall posts	Facebook is used by diabetic patients & friends / members to share personal clinical information, request disease specific guidance & feedback, & for emotional support	Facebook provides a platform for people living with diabetes to report personal experiences, ask questions & receive direct feedback<br><br>No accountability or checks for authenticity of information or sharing of personal data	Facebook enables people living with diabetes to share their experiences, ask questions & receive direct feedback
Hu & Sundar (2010) [51]	Full-factorial online experiment (quantitative)	Blog	To examine the direct & combined influences of original sources (doctors vs. laypersons) & selecting sources (web sites vs. bulletin boards vs. blogs vs. personal home pages	555 undergraduate students	Online questionnaire	Respondents were more likely to take action based on a website than a blog or a personal homepage – this was dictated by level of perceived gate keeping & completeness of information	New online source typology was developed including social media	Blogs are an online source of health information

			vs. internet) on perceived credibility of –& behavioral intentions towards – health information					
Hwang et al. (2010) [52]	Mixed methods	Online weight loss communities - SparkPeople	To determine whether an online version of the weight management support inventory is appropriate for measuring social support among members of Internet weight loss communities	206 members of an internet weight loss community (Survey n=193; Interviews n=13)	Questionnaire Telephone interviews Forum messages	Participants frequently used forums (56.8% reading messages; 36.1% replying to messages; 18.5% posting messages to start a discussion relating to weight loss on a daily or more frequent basis)<br><br>Encouragement & motivation were major social support themes, mentioned at least once by 87.6% of survey respondents, followed by information (58.5%), & shared experiences (42.5%)	Internet weight loss communities provide a forum for social support to aid weight loss	Internet weight loss communities provide a forum for social support to aid weight loss
Kim & Kwon (2010) [53]	Secondary data analysis using the National Cancer Institute's 2005 Health information national trends Survey (HINTS) (quantitative)	Online social networks, e.g. online support groups	To uncover how cancer e-patients differ from other cancer information seekers in terms of their socioeconomic background, social networks, information competence, & selection of cancer information sources	5,586 members of the US general adult population	Computer-assisted telephone interviews & web-based interviews	Cancer e-patients were older & talked about their health more frequently compared to online information seekers without cancer<br><br>E-patients preferred information from their doctors over the Internet, & used the Internet as their primary source	There is a need for better design & delivery of health information literacy programs for cancer e-patients	Online social networks sites are used by cancer e-patients to access information form their doctor
Kontos et al. (2010) [54]	Secondary data analysis using the National Cancer Institute's 2007 Health information national trends Survey (HINTS) (quantitative)	SNS e.g. Facebook	To identify potential communication inequalities in social networking site use among a representative sample of U.S. adults & to examine the association between SNS use & psychological well-being	3,582 American adults	Secondary data analysis from telephone interviews	35% of online adults reported using a SNS in the past 12 months<br><br>No significant differences in SNS use by race / ethnicity, or socioeconomic position<br><br>Younger age (18-24 years) was the most significant predictor of SNS uses whereas being married or having cancer decreased odds of SNS use	The absence of inequalities in adults SNS use across race / ethnicity & socioeconomic position offers support for continued use of social media to promote public health efforts	SNS should be used for public health communication as there is an absence of inequalities across race / ethnicity in their use
Lagu (2010) [55]	Descriptive (qualitative content	Physician-rating websites	To describe the structure & content of physician-rating	33 physician-rating websites, 190 reviews for	Content analysis of website reviews	88% reviews were positive; 6% negative; 6% neutral	Physician-rating websites allow patients to provide feedback & obtain information about physician	Physician rating websites allow patients to provide feedback on physician

	analysis)		websites & to assess the extent to which a patient might find them valuable	81 physicians			performance	performance
Lariscy et al. (2010) [56]	Survey (quantitative)	Social Networking media	To examine adolescents' reported health learning about a variety of health concerns through differing media, including online social media, across racial, gender & geographical lines	452 adolescents	Questionnaire	Adolescent's most reported health information sources from television, followed by radio print, Internet & social networking sites<br><br>Internet & online social media are more important for rural adolescents than urban ones	Social media may have potential to be used by communication professionals to connect with adolescents	Social media may have potential to be used by communication professionals to connect with adolescents
Lo et al. (2010) [57]	Descriptive (quantitative)	YouTube	To see how epilepsy is being perceived (on YouTube)	Top 10 epilepsy videos on YouTube<br><br>(top 10 hits identified)	YouTube videos	The top 10 video are being viewed 3200 times daily<br><br>All videos showed statistically significant differences in at least one variable: empathy or knowledge ($p < 0.001$)	Importance of recognizing the significant attributes of videos that engage viewers	Video-sharing websites, like YouTube have potential to correct misinformation or stigma surrounding epilepsy
Nordfeldt et al. (2010) [58]	Qualitative	Web 2.0 portal (tailored to young patients with type 1 diabetes)	To explore patient's & parent's attitudes toward a local Web 2.0 portal tailored to young patients with type 1 diabetes & their parents, with social networking tools such as message boards & blogs, locally produces self-care & treatment information, & interactive pedagogic devices	24 participants – 16 mothers; 3 fathers; 5 young diabetes patients (11-18 years)	Analysis of essays (written by mothers, fathers & young diabetes patients)	3 main categories of portal user's attitudes "management tool", "the generator" & "the gatekeeper"	Web 2.0 services have great potential for supporting parents & patients with type 1 diabetes by enhancing their information retrieval & disease management but needs to be maintained & updated	Web 2.0 portal services show potential for supporting parents & patients with type 1 diabetes by enhancing their information retrieval & disease management
Orizio et al. (2010) [59]	Descriptive (qualitative content analysis)	Online social networks focused on diseases	To analyze the existing online social networks dedicated to health issues	41 online social networks focused on diseases	Online social networks focused on diseases	12.2% showed the name of the author or operating organization, 87.8% provided a way of contacting the website<br><br>4.9% published statistical information about patients registered with the SNS	Data showed a large variety of health SNS<br><br>Quality of online health information is raised, as is the use of personal data & privacy issues	SNS provided health information
Rice et al.	Survey	Social	To examine the	201 homeless	Survey	Increased time online & recent	Homeless youth need more access to the	Online SNS can be used by

(2010) [60]	(quantitative)	networking sites such as Facebook & MySpace	association between sexual health & internet use, including social networking sites such as MySpace & Facebook	adolescents at risk of contracting HIV/ aids		engagement in exchange sex were positively associated with online partner seeking<br><br>Youth connected to street-based peers online were more likely to practice exchange sex, whereas youth connected to home-based peers were more likely to report a recent HIV test & less likely to practice exchange sex	Internet as access facilitates connecting with family & home-based peers whose presence may reduce sexual risk taking	homeless adolescents to connect with family & home-based peers whose presence may help reduce sexual risk taking
Sanford (2010) [61]	Qualitative	Blogs	Explores the use of weight loss blogs by the morbidly obese	50 bloggers who need to lose 100 pounds or more, had been blogging for at least 3 months about weight loss & had not had bariatric or lap b& surgery at the time the surveys were administered	Qualitative survey	Blogging provides invaluable computer-mediated social support as it allows for empathy, ensures accountability to others, provides venting & advice seeking, & shares validation of the weight loss experience	Blogging can provide social support & information & advice for weight loss	Blogging can provide social support & information & advice for weight loss
Scanfeld et al. (2010) [62]	Descriptive (qualitative content analysis)	Twitter	Review Twitter status updates mentioning "antibiotics" to determine overarching categories & explore evidence of misunderstanding or misuse of antibiotics	971 Twitter status updates	Content analysis of Twitter status updates	Status updates were categorized into 11 groups: general use; advice / information; side effects / negative reactions; diagnosis; resistance; misunderstanding & or misuse; positive reactions; animals & other; wanting / needing; cost	Twitter offers means of health information sharing	Twitter may be used to identify misunderstanding of health information, promote positive behavior, disseminate valid information & to gather real-time data which can be leveraged in public health communication
Selby et al. (2010) [63]	Explorative & descriptive (qualitative content analysis)	Online smoking cessation support group	To compare the characteristics of smokers who post in an online smoking cessation support group with smokers who do not post, conduct a qualitative analysis of discussion board content, &	Data from Stopsmokingcenter.net version 5.0, a web-assisted tobacco intervention<br><br>(from 16,764 site users)	Content analysis of data from Stopsmokingcenter.net version 5.0, a web-assisted tobacco intervention	15% (2562) made at least one post in the online social support network; 25% of first posts received a response from another member within 12 minutes, 50% within 29 minutes	Peer responses to new users were rapid, indicating that online social support networks may be particularly beneficial to smokers requiring more immediate assistance with their cessation attempt<br><br>This function may be especially advantageous for relapse prevention	Online smoking support cessation groups provide peer support that may help smokers to quit, & may help prevent relapse

			determine the time it takes for new users to receive feedback from existing members or moderators					
Tian (2010) [64]	Descriptive (qualitative content analysis)	YouTube	To examine the content of & audience response to organ donation videos on YouTube	355 YouTube videos – 1,634 entries with active links	YouTube videos	Most videos were positive about organ donation (more so than traditional media)	YouTube videos provided information on organ donation	YouTube videos provided information on organ donation
Wicks et al. (2010) [65]	Cross-sectional online survey (quantitative)	PateintsLike Me	To describe the potential benefits of PatientsLikeMe in terms of treatment decisions, symptom management, clinical management & outcomes	1323 members from six PatientsLikeMe communities	Online survey	Benefits - learning about a symptom they had experienced / with information about medication / understanding the side effects of their treatments<br><br>42% reported finding someone who had helped them understand what it was like to take a specific treatment for their condition	Members of the community reported a range of benefits & these may be related to the extent of the site use	PatientsLikeMe is used by patients to find & share information on their disease, including symptoms, treatment & medication
Adrie et al. (2011) [66]	Survey (quantitative)	Websites enabling online health companion contact, e.g. discussion forum or SNS	To understand, motivation, technology & effects of online health companion contact concerning chronic diseases	353 website users	Online questionnaire	Obtaining information was the key motivation for applying on-line health companion contact<br><br>Other factors are the possibility to share experiences with other patients, to find recognition, & understanding & to meet new people<br><br>Barriers included privacy concerns, negative stories & concerns regarding the quality of information	Online health companion contact can increase the quality of life & self-management because respondents perceived to be better informed, better able to accept their disease, better deal with their situation & to receive an increased amount of social support	Websites with message posting facilities can be leveraged for social support by patients with chronic diseases
Baptist et al. (2011) [67]	Survey (quantitative)	Electronic media (including email, social media such as Facebook, & text messaging)	To survey collected demographic information, use of electronic media, interest in using electronic media to receive asthma information, & interest in using electronic media to communicate	145 asthma patients, 12-40 years of age	Survey	Email was the most preferred method to receive asthma information & to communicate with a physician<br><br>There was some interest in using Facebook or text messaging, whereas MySpace & Twitter had minimal interest	Social media sites like Facebook may be appropriate for certain asthma patients	Social media sites such as Facebook may be appropriate to communicate with some asthma patients

			with a health care provider about asthma					
Bender et al. (2011) [68]	Descriptive (qualitative content analysis)	Facebook	To characterize the purpose, use, & creators of Facebook groups related to breast cancer	620 breast cancer groups on Facebook, a total of 1,090,397 members	Content analysis of breast cancer groups on Facebook	Groups were created for fundraising (44.7%), awareness (38.1%), product or service promotion related to fundraising or awareness (9%), or patient/caregiver support (7%)	Facebook groups have become a popular tool for awareness-raising, fundraising, & support-seeking related to breast cancer attracting over one million users	Facebook groups are a tool for awareness-raising, fundraising, & support-seeking for breast cancer
Bosslett et al. (2011) [69]	Survey (quantitative)	Online social networks (OSNs)	To quantify the use of OSNs, patient interactions within OSNs, & attitudes toward OSNs among medical students (MS), resident physicians (RP), & practicing physicians (PP)	1004 MS, 1004 RP, & 1004 PP between February & May 2010.	Random, stratified mail survey	93.5% MS, 79.4% RP, & 41.6% PP reported usage of OSNs	Patient–doctor interactions take place within OSNs, & are more typically initiated by patients	SNS may be used by patients to interact with physicians online
Chou et al. (2011) [70]	Descriptive (qualitative)	YouTube	To provide an in-depth description of authentic personal cancer stories	35 YouTube videos identified by the search term “cancer”	YouTube videos	Sub narrative on the cancer diagnosis was present in 86% of stories<br><br>Narratives were characterized by dramatic tension, emotional engagement, markers of the loss of agency or control, depersonalized reference to the medical personnel, & the unexpectedness of a cancer diagnosis	YouTube is used to exchange personal cancer stories & has potential for narrative based health communication	YouTube is used to exchange personal cancer stories & has potential for narrative based health communication
Doing-Harris & Zeng-Treitler (2011) [71]	Qualitative	Social network data from PatientsLike Me	To create a computer assisted update (CAU) system that works with live corpora to identify new candidate terms for inclusion in the open access & collaborative (OAC) Consumer Health Vocabulary (CHV)	CAU system consisting of three main parts: a Web crawler & an HTML parser, a candidate term filter that utilizes natural language processing tools including term recognition	The CAU system was applied to the health-related social network website PatientsLikeMe	The CAU system identified 88,994 unique terms 1- to 7-grams in 300 crawled PatientsLikeMe.com webpages<br><br>The manual review of the crawled webpages identified 651 valid terms not yet included in the OAC CHV or the Unified Medical Language System (UMLS) Metathesaurus, a collection of vocabularies amalgamated to form an ontology of medical terms, (i.e., 1 valid term per	The CAU system is effective for generating a list of candidate terms for human review during CHV development	A CAU system can be used to generate a list of candidate terms for human review during CHV development

				methods, & a human review interface		136.7 candidate n-grams)		
Dowdell et al. (2011) [72]	Survey (quantitative)	SNS	To survey middle school, high school, college student & sexual offenders about their use of social networking sites in order to better focus education & prevention efforts from healthcare providers	4,231 participants (404 middles school students<br><br>2,077 high school students<br><br>1,284 college students<br><br>466 adults who had committed an Internet or hands-on sexual offence)	Questionnaire	Offenders & students both frequent SNS - offenders reported they preferred MySpace & students Facebook<br><br>Nearly 2/3 of offenders reported that they had initiated the topic of sex in their first chat session, & they preferred to communicate with teenage girls online	SNS present new & complex issues relating to safety & privacy of adolescents<br>Possible nurse initiated solutions include designing technologies & educational programs to help identify suspicious online behaviors	SNS are used by sexual offenders to communicate with young people online<br><br>SNS may be leveraged for health education messages about online safety
Egan & Moreno (2011a) [73]	Descriptive (qualitative content analysis)	Facebook	To identify self-reported stress & associated conditions among college students on Facebook	300 undergraduate freshmen at a large Midwestern state university	Content analysis of Facebook profiles	Stress references were displayed on 37% of the profiles, weight concerns on 6%, depressive symptoms on 24%, & alcohol on 73%<br><br>Display of stress references was associated with female sex, weight concerns, & depressive symptoms	Facebook may be a useful venue to identify students at risk for stress-related conditions & to disseminate information about campus resources to these students	College freshmen frequently display references to stress on Facebook profiles with prevalence rates similar to self-reported national survey data
Egan & Moreno (2011b) [74]	Descriptive (qualitative content analysis)	Facebook	To identify references to alcohol on college males' Facebook profiles	225 undergraduate males Facebook profiles	Content analysis of Facebook profiles	Alcohol references were present on 85.3% of the profiles & prevalence of alcohol was similar across each undergraduate grade<br><br>An increase in number of Facebook friends was associated with an increase in displayed alcohol references	Widespread alcohol displays on Facebook may influence social norms & cause increases in male college students' alcohol use	Alcohol displays are common on Facebook
Friedman et al. (2011) [75]	Descriptive (qualitative content analysis)	Social bookmarking websites	To examine the content of Prostate Cancer (PrCA) prevention & screening information being posted &/ or tagged on social	127 webpages	Content analysis of Webpages	Majority of content was from news websites (48.9%) & blogs (37.8%)<br>PrCA screening was mentioned on 95.3% of pages; 30.7% discussed the prostate-specific antigen test<br><br>Less than half (43.8%) mentioned	PrCA content is inconsistent on Web 2.0 sites	Web 2.0 sites are used to post PrCA information

			bookmarking websites.			current screening guidelines		
Frimmings et al. (2011) [76]	Case Study (quantitative)	Facebook group site	To survey student reflections of a social media experience	127 students split into two groups: 1.learners (N=92) 2.preservice health & fitness professionals (N=35)	Survey	51.1% of the learner group reported long-term use of a social media site may benefits their fitness routine<br><br>52.9% of the preservice health & fitness group reported learning from peers	University is a good setting to share health & fitness knowledge through social media	Social media sites may be used effectively to communicate health & fitness knowledge in a university setting
Frost et al. (2011) [77]	Descriptive (qualitative content analysis)	PatientsLikeMe	To examine the illustrative cases of amitriptyline & modafinil – drugs commonly used off-label	1948 treatment histories for modafinil<br><br>1394 treatment reports for amitriptyline reported across five PatientsLikeMe communities	Content analysis of Treatment histories & reports on PatientsLikeMe	Most members reported taking the drug for off-label uses<br><br>Minority reported using modafinil or amitriptyline for its approved purpose	PatientsLikeMe offers unique real-time approach to understand utilization & performance of treatments across many conditions & can provide a new source of evidence about secondary uses & potentially identify targets for treatments to be studied systematically in traditional efficacy trials	PatientsLikeMe can be used to understand how treatments are being used across a range of conditions
Gajaria et al. (2011) [78]	Ethnographic content analysis (qualitative)	Facebook	To examine what youth think about having ADHD by collecting data in a naturalistic setting- a popular SNS	Text from 25 English language Facebook groups with “ADHD” in the tile & created or administered by someone with a current high school or university affiliation & at least 100 members	Content analysis of ADHD Facebook groups	The main theme identified was the collective construction of a positive group identity<br><br>Facebook groups functioned like electronic support groups, with members providing support to one another & sharing experiences & information, including advice about medication<br><br>Many jokes about ADHD stereotypes were posted	Facebook was used by youth as a supportive environment to develop a group identity & reject negative aspects of common stereotypes related to young people with ADHD	Facebook can provide an electronic support group function for young people with diseases or learning needs e.g. ADHD
Garcia-Romero et al. (2011) [79]	Pilot teledermatology study (quantitative)	Facebook	To evaluate: 1. The quality of the digital images on Website & the feasibility of making a diagnosis	44 dermatologic patients	Dermatologic patients took digital pictures & uploaded onto the website, which	A GP used Facebook to send consults to a dermatology department at a general hospital - 44 patients were seen & given treatment	Majority of patients (75%) benefited from the diagnoses & treatments, avoiding unnecessary expenses or transportation to urban areas	Facebook can be used to provide dermatology consults, thus saving unnecessary expenses or transport to urban areas

			through them.<br>2. The efficacy of the treatments indicated<br>3. Impact of the project on patients' benefit<br>4. Identifying areas of opportunity & needs to improve the project & expand it in the future		were analyzed by dermatologists	Simple-to-treat, common skin diseases & rare congenital diseases that require genetic counselling & more complex treatment were identified		
Hanson et al. (2011) [80]	Survey (quantitative)	Social media applications e.g. Facebook, MySpace	To explore social media use & factors that determine acceptance of social media use among health educators	503 certified health education specialists	Online survey	Health educators most commonly use SNS, podcasts & social media sharing sites within their organisations<br><br>Social influence & behavioural expectancy were positively associated with increased behavioural intentions to use social media for health promotion	Many health educators are using social media & intentions to use in practice is associated with social influence & performance expectancy	Social media is being used by many health educators
Heavillin et al. (2011) [81]	Descriptive (qualitative content analysis)	Twitter	To evaluate whether Twitter users broadcast information relating to dental pain & assess the content of the information being communicated	772 Tweets from a random sample of 1000 Tweets randomly selected from 4859 Tweets over 7 non-consecutive days	Content analysis of Tweets	83% of Tweets were primarily categorised as a general statement of dental pain; 22% as an action taken or contemplated; 15% as describing an impact on daily activities<br><br>Among the actions taken or contemplated 44% reported seeing a dentist; 43% took an analgesic or antibiotic; 14% sought advice from the Twitter community	Twitter users widely share health information relating to dental pain, including actions taken to relive pain & the impact of pain	Twitter is used by members of the public to post information about dental pain<br><br>Twitter may provide an opportunity for dental professionals to disseminate health information
Jent et al. (2011) [82]	Descriptive (quantitative)	Facebook	To examine the prevalence with which healthcare providers use a social media site (SMS) account (e.g., Facebook), & the extent to which they use SMSs in clinical practice, & their decision- making	109 pediatric faculty & trainees from a medical school campus	3 Questionnaires:<br>1.Demographic information<br>2.SMS history form<br>3.SMS fictional profile	Small percentage of trainees reported a personal history of conducting an Internet search (18%) or an SMS search (14%) for a patient - no faculty endorsed a history of conducting searches for patients	Facebook is used by a small percentage of medical trainees to conduct an Internet search for a patient	Facebook is used by a small percentage of medical trainees to conduct an Internet search for a patient

			process after accessing patient information from an SMS					
Kadry et al. (2011) [83]	Quantitative content analysis	Physician rating websites with user generated content	To determine the most frequently visited physician-rating websites with user-generated content, (2) evaluate the available information on these websites, & (3) analyze 4999 individual online ratings of physicians	10 most frequently visited online physician-rating sites	Content analysis of online physician-rating sites	The average rating was 77 out of 100 for sites using a 100-point scale<br><br>The patient's single overall rating of the physician correlated with the other dimensions of care that were rated by patients for the same physician (Pearson correlation, r = .73, P < .001).	Most patients give physicians a favorable rating on online physician-rating sites<br><br>A single overall rating to evaluate physicians may be sufficient to assess a patient's opinion of the physician	Physician rating websites are being used by patients to provide feedback on doctor's performance following consultations
Kishimoto & Fukushmima (2011) [84]	Web-based cross-sectional survey (quantitative)	Anonymous web communities including social media, e.g. Twitter	To investigate the status of researching drug information online, & the type of Internet user who uses anonymous web communities	10875 male & female Internet users aged 16 yrs & over	Survey	47% respondents reported previously searched the Internet for drug information & had used a range of resources including drug information search engines & Twitter	Results revealed the profiles of Internet users who researched anonymous drug websites	Anonymous web communities are used to search for drug information
Kukreja et al. (2011) [85]	Survey (quantitative)	Facebook & Twitter	To define current use patterns of Facebook & Twitter among pharmacy preceptors & assess perceptions regarding use of social media within professional practice	155 pharmacist registered as advanced pharmacy practice experience (APPE) preceptors	Online survey	Approximately 60% of respondents used a Facebook account & 9% used a Twitter account<br><br>Respondents were willing to complete continuing education (CE) credit (46%) using social media, & were interested in following professional organizations (39%) on social media;<br><br>Majority were not interested in obtaining drug or disease-state information, identifying employment opportunities, or participating in clinical discussion forums via social media	Despite the growing status of social media across multiple disciplines, the majority of pharmacy preceptors surveyed were not willing to use these venues in professional practice	Facebook is widely used by pharmacy preceptors for personal activities
Lariscy et al. (2011) [86]	Qualitative	Information sources e.g. MySpace	To identify health concerns & health behaviors, to	42 7th grade students in 2 poor & near-	Focus groups	Online social media provide a parasocial experience	Social media should be examined as future components of primary socialisation theory	Online social media are used by some young people to access health information

			understand why some concerns are more worrisome than others, & to learn what information sources are relied on for a young age cohort- 7th grade students	poor school districts: 1 rural boys 13 rural girls 10 urban boys 8 urban girls				
Lau (2011) [87]	Survey (quantitative)	Web 2.0 tools, e.g. blogs, wikis	To investigate how Web 2.0 tools can be applied for knowledge sharing, learning, social interaction, & the production of collective intelligence in the nursing domain & to investigate what behavioral perceptions are involved in the adoption of Web 2.0 tools by nurses	388 nurses	Questionnaire	Intention toward using Web 2.0 tools was positively correlated with usage behavior (*r* = .60, *P* < *.05)*<br><br>Behavioral intention was positively correlated with attitude (r = .72, *P* < .05), perceived behavioral control (*r* = .58, *P* < .05), & subjective norm (*r* = .45, *P* < *.05*)	Identified behavioral perceptions may further health policy makers' understanding of nurses' concerns regarding & barriers to the adoption of Web 2.0 tools & enable them to better plan how to implement Web 2.0 tools for knowledge sharing, learning, social interaction, & the production of collective intelligence	Web 2.0 tools can be used for knowledge sharing, learning, social interaction, & the production of collective intelligence in the healthcare arena
Liang & Scammon (2011) [88]	Case study (qualitative)	SNS	To analyze discussion threads posted to an obesity support group on a popular health SNS	An obesity support group on a popular health SNS	Discussion threads posted to an obesity support group on popular SNS	SNS provides informational & emotional support that helps support seekers actively cope with health-related problems & strains	Support seekers participation in SNS provides opportunity for learning	SNS provide emotional & informational support to help users cope with health-related problems
Lord et al. (2011) [89]	Survey (quantitative)	Facebook	To assess the feasibility of implementing a survey study about prescription medication misuse with college students on Facebook & identify the characteristics, motivations, beliefs, & attitudes associated with experimental versus regular misuse of prescription opioid analgesic medications.	527 college students	Survey on Facebook	A 2-week random advertisement post on Facebook yielded an above average response to online drug use survey	Facebook has the potential to connect with college aged populations about their drug use	Facebook has may reach college populations, e.g. online marketing campaigns
Morturu &	Experimental	Wikipedia &	To addressees the trust	Wikipedia	Experimental	This approach is effective & can be	This approach can be easily adapted &	Wikipedia & Daily Strength

Liu (2011) [90]	analysis (2 case studies, quantitative)	Daily Strength	assessment problems & proposes an relevant solution that is not restricted to a specific social media application	Daily Strength - data from an Autism-Autism-Spectrum support group	analysis using their trust assessment tool to analysis Wikipedia & Daily Strength	adapted to disparate social media applications with ease & models sound	applied across social media & is its most distinguishing characteristic	provide health information
O'Dea & Campbell (2011) [91]	Cross-sectional survey (quantitative)	Online social networks	To explore the potential of SNS as an avenue for peer mental health support	74 secondary students	Survey	Online SNS were used regularly by 82% of participants<br><br>47% believed these sites could help with mental health problems	Online SNS may be able to connect young people with others experiencing mental health difficulties	SNS offer social support & information for those experiencing mental health problems
Omurtag et al. (2011) [92]	Cross-sectional study (quantitative)	Social networking websites	To study & describe the use of social networking websites among Society for Assisted Reproductive Technology (SART) member clinics	384 SART member clinics in a university based practice	Prevalence of social networking websites among SART member clinics	96% of SART member clinics had a website & 27% hosted a link to a SNS<br><br>Majority of posts dealt with the clinic by advertising (29%) or providing (29%) information to the viewer or offering patient support (19%)	Clinics use SNS to advertise & provide information to patients	Fertility clinics use SNS to advertise & provide information to patients
Rajagopalan et al. (2011) [93]	Comparative study (quantitative)	Wikipedia	To compare the coverage, accuracy, & readability of cancer information on Wikipedia with those of the patient-orientated National Cancer Institute's Physician Data Query (PDQ) comprehensive cancer database.	Wikipedia & PDQ comprehensive cancer database for each of the ten cancer types	Appraisal forms Validated Flesch-Kincaid grade level	Inaccuracies were rare for both Web sites<br><br>PDQ was significantly more readable than Wikipedia: Flesch-Kincaid grade level 9.6 versus 14.1	Although Wiki resource had similar accuracy & depth as the professionally edited database, it was significantly less readable	Wikipedia provides relatively reliable cancer information
Ralph et al. (2011) [94]	Mixed methods (surveys; focus groups, interviews)	MySpace	To explore the potential for using social networking sites (SNS), specifically MySpace, to connect	Youth aged 14-19 years, living in low income communities in California	Surveys<br>Focus groups (with youth)<br>Interviews (with clinic staff)	High levels of overall Internet access, frequent use of SNS, & experience in searching for health information online	SNS has strong potential for reaching diverse youth with critical health information when implemented as part	SNS have strong potential for reaching diverse youth with critical health information, as part of a wider strategy

			youth to sexual health services	Sexual health clinic staff		Disparities in frequency & location of Internet access by race/ethnicity, & hesitancy among some youth to join a clinic's online social network, may affect limit success	of a comprehensive outreach strategy	
Sajadi & Goldman (2011) [95]	Descriptive (qualitative content analysis)	Facebook Twitter YouTube	To evaluate & catalogue results of a social media search for information on incontinence	The first 30 results for "incontinence" on Facebook, Twitter & YouTube	Content analysis of results for "incontinence" on Facebook, Twitter & YouTube	Facebook – 4 results (13%) were informative; 14 (47%) had no usable information<br>Twitter- 18 (60%) linked to incontinence related healthcare information<br>YouTube – 14 (47%) of videos were informational; 3% were not useful	Social networks have insufficient useful incontinence content, especially from healthcare professionals & incontinence organisations<br><br>Medical professionals & societies use these avenues to reach & educate patients	Facebook, Twitter & YouTube provide limited information on incontinence
Salthe & Khandelwal (2011) [96]	Descriptive (qualitative content analysis)	Twitter	To measure the spatio-temporal sentiment towards a new vaccine	Publicly available data from 101,853 users of online social media collected over a time period of almost six months to measure the spatio-temporal sentiment towards a new vaccine	Content analysis of 477,768 collected tweets	Information flows more often between users who share the same sentiments<br><br>Most communities are dominated by either positive or negative sentiments towards the novel vaccine	Online social media provide unprecedented access to data allowing for inexpensive & efficient tools to identify target areas for intervention efforts & to evaluate their effectiveness	Twitter can be used to identify target areas for intervention efforts & to evaluate their effectiveness
Selkie et al. (2011) [97]	Mixed methods	SNS	To determine adolescents views regarding how new technologies could be used for sexual health education	Adolescent 15-19 year olds	Focus groups<br><br>Surveys	Adolescents were enthusiastic about using online / SNS resources for sex education	Online SNS resources have potential for sex education & adolescents are open to interacting with sexual health educators on social networking sites	SNS have potential for communicating with adolescents to provide sexual health education
Setoyama et al. (2011) [98]	Exploratory, descriptive web-based survey (quantitative)	Online breast cancer communities	To explore the differences in peer support received by lurkers & posters in online breast cancer communities & examine the effects of such support on both groups mental health	253 posters / lurkers in online breast cancer communities	Online questionnaire	5 support functions were identified for both posters & lurker: emotional support/helper therapy, emotional expression, conflict, advice, & insight/universality<br><br>Emotional support/helper therapy & emotional expression were significantly higher among posters (P<0.05)	Posters felt they received more benefits from online communities than lurkers<br><br>Lurkers were found to gain a certain amount of peer support through online communities	Online communities provide peer support & may be beneficial to breast cancer patients' mental health

Shah & Robinson (2011) [99]	Descriptive (qualitative content analysis)	Blogs	To elicit patients' perspectives & experiences regarding PT/INR self-testing using portable coagulometer devices	246 blog postings by 108 patients on oral anticoagulation therapy (OAT)	Content analysis of patient's blog postings on OAT	Patient benefits reported were time saved, personal control, choice, travel reduction, cheaper testing, & peace of mind	Patients' blogs on PT/INR testing provide insightful information that can help in understanding the nature of the experiences & perspectives of patients on self-testing of OAT	Patients' blogs provide information which can help us understand the experiences of those on self-testing of OAT
Shrank et al. (2011) [100]	Descriptive (qualitative content analysis)	Most commonly used SNS focused on diabetes e.g. Facebook	To explore characteristics of online diabetes SNS communities, & better inform physicians & patients about the choice available to them	15 SNS focused on diabetes	Content analysis of SNS focused on diabetes	Level & type of health professional participant varied widely across site<br><br>Physicians were available to answer questions in 33% of sites, but scanned posts & only responded to 7% of postings	Online SNS have an increasing role in health promotion (as time for telephone consultations decreases & the a web savvy population ages)	SNS facilitate discussion of diabetes management between patients, & patients & health professionals
Signorini et al. (2011) [101]	Descriptive (quantitative)	Twitter	To examine the use of information embedded in the Twitter stream to (1) track rapidly evolving public sentiment with respect to H1N1, & (2) track & measure actual disease activity	2 data sets: 1.951,697 tweets 2.4,199,166 tweets	Analysis of tweets about H1NI	Estimates of influenza like illness derived from Twitter chatter accurately track reported disease levels<br><br>Twitter traffic can be used descriptively i.e. to track users' interest & concerns, & but also to estimate disease activity in real time i.e. 1-2 weeks faster than current practice enables	Twitter-based surveillance efforts may provide & important & cost-effective supplement to traditional disease-surveillance systems<br><br>Twitter data can be used to test the effectiveness of public health messaging & health campaigns	Twitter can be used to track & monitor disease outbreak, & to test the effectiveness of public health messaging & health campaigns
Turner-McGrievy & Tate (2011) [102]	Randomized intervention (quantitative)	Twitter, Apps, Podcasts	To examine whether a combination of podcasting, mobile support communication, & mobile diet monitoring can assist people in weight loss	96 overweight adults Weight loss intervention delivered via 2 groups: 1.Podcast only 2. Podcast & mobile	Questionnaires	Weight loss did not differ by group at 6 months: mean –2.7% (SD 5.6%) Podcast+Mobile, n = 47; mean –2.7% (SD 5.1%) Podcast, n = 49; *P* = .98	Minimally intensive weight-loss intervention can be delivered via podcast, but prompting & mobile communication via Twitter & monitoring app without feedback did not enhance weight loss	Twitter (via mobile communication) without feedback can be used for delivering weight loss interventions but is not effective
Usher et al. (2011) [103]	Survey (quantitative)	Web 2.0	To identify the main types of social media (Web 2.0) technologies utilized by Australian AHPs to	935 participants from 8 of Australia's major AHPs	Online survey	Health Professionals are interacting with Web 2.0 technologies for personal use but are failing to implement such technologies throughout their health professions	Web 2.0 technologies have potential for health communication practice & health promotion, but are not currently being used by AHPs to deliver health care	Web 2.0 technologies have potential for health communication practice & health promotion

			deliver early twenty-first century health care, practice promotion & personal communication.			to deliver health care & are willing to undertake online educational courses		
Van Uden-Kraan (2011) [104]	Survey (quantitative)	Web 2.0 (peer-to-peer support)	To explore factors that facilitate or impede engagement in face-to-face & online peer support, using the Theory of Planned Behaviour	679 patients being treated for arthritis, breast cancer, or fibromyalgia	Questionnaire	4% (30/679) respondents had contact with peers via the Internet in the past year<br><br>Patients were more positive about face-to-face peer support than about online peer support ($P < .001$)	Minority of patients seem to be interested in organized forms of peer contact (either online or face-to-face)<br><br>Patients were more positive about face-to-face contact than about online contact	Web 2.0 can be used by patients for peer support
Veinot et al. (2011) [105]	Qualitative (focus groups)	Information & communicatio n technologies (ICTs) e.g. MySpace	To examine how youth use ICTs in order to socialize with others, & how this aligns with their communication about sexuality & HIV/STIs	94 youth from one economically depressed city with a high STI/HIV prevalence	12 focus groups	Key themes (distraction, diversification, dramatization, danger management & dialogue) suggest that young people in this community use technology in similar ways to young people in other settings but they also exhibit unique traits arising from the characteristics of their local communities, e.g. the relatively high prevalence of HIV / STIs where they live	Need for online interventions that address young people's need for safety, while creating online experiences that are as interesting as the "drama " that both engages & repels them	Online methods may effectively reach young people to communicate their need for safe sexual practices
Weitzman et al. (2011) [106]	Observational study (quantitative)	Facebook-like environment	Tested the willingness of an online diabetes community to share data for public health research by providing members with a privacy-preserving social networking software application for rapid temporal-geographic surveillance of glycaemic control.	1,136 members of an international online diabetes SNS	SN-mediated collection of cross-sectional, member-reported data	Sharing was high with 81.4% of users permitting data donation to the community display<br>34.1% of users also displayed their A1cs on their SN profile page<br>Users selecting the most permissive sharing options had a lower average A1c (6.8%) than users not sharing with the community (7.1%, p = .038)	Success within an early adopter community demonstrates that online SNs may comprise efficient platforms for bidirectional communication with & data acquisition from disease populations	SNSs may be efficient platforms for bidirectional communication with, & data acquisition from disease populations
Young & Rice (2011)	Survey (quantitative)	SNS	To evaluate associations between	201 homeless youth accessing	Survey	Using online social networks for partner seeking is associated with	Online SNS & the topics discussed on these networks can potentially increase	SNS could be used to develop sexual health

[107]			online social networking & sexual health behaviors among homeless youth	services		increased sexual risk behaviours<br><br>Online social network usage is associated with increased knowledge & HIV/STI prevention among homeless youth	& decrease sexual risk behaviors depending on how the networks are used	services & interventions & could reduce sexual risk behaviors
Fernandez-Luque et al. (2012) [108]	Link analysis (quantitative)	YouTube videos	To explore approaches for extracting metrics about authoritativeness in online health communities & how these metrics positively correlate with the quality of the content	Two different experiments, health consumers provided 427 ratings of 17 YouTube videos on diabetes & professionals gave 162 ratings of 23 YouTube videos on diabetes<br><br>2 professionals reviewed 30 diabetes channels	A metric, called HealthTrust (designed for the study), that estimates the trustworthiness of social media content (e.g. blog posts or videos) using link analysis	HealthTrust's filtering allowed 3 bad channels (15%) versus 8 (40%) on the YouTube list<br><br>Misleading & graphic videos (e.g., featuring amputations) were more commonly found by YouTube Search than by searches based on HealthTrust<br><br>However, some videos from trusted sources had low HealthTrust scores	HealthTrust showed promising results when used to retrieve diabetes content from YouTube<br><br>Social network analysis may be used to identify trustworthy social media in health communities	YouTube video searches can be filtered e.g. to remove misleading results, using a metric such as HealthTrust
Marcus et al. (2012) [109]	Qualitative	Blogs	To move beyond past research by using an innovative qualitative research method of analyzing the blogs of young adults (18–25 years of age) with mental health concerns to understand their experiences	8 blogs: authored by 18- to 25-year-olds; frequently updated & viewed; authored by 2 men & 6 women who had a variety of mental health problems; & written between Aug 2008 - Feb	Grounded theory & consensual qualitative research methods	2 core categories emerged from the qualitative analysis of the bloggers accounts: I am powerless (intrapersonal) & I am utterly alone (interpersonal)	One reason young adults do not seek care might be that they view the mental health system negatively & feel disconnected from these services	Blogs provide an account of the experiences of young people (18-15 years) with mental health concerns

				2009				
O'Grady et al. (2012) [110]	Mixed methods (survey, interview, usage tests)	Web site message forums	To investigate how structured (pre-determined) & unstructured (user-generated) tags & tag clouds with a multiple word search feature are used by participants to assess credibility of messages posted in online message forums	22 participants, already using web sites message forums for disease self-management	A demographic & computer usage survey<br><br>A series of usability tests<br><br>Interview	Less than one quarter of participants had seen or used tags or a tag clouds<br><br>Some participants were tagging the information for their own subsequent use, whereas others viewed this process from the perspective of others: they tagged the content in ways that they thought other users would find beneficial	Many participants in this study focused on assessing whether the information was relevant to their current circumstances, after which they would proceed to determine its credibility by corroborating with other sources	SNS are used to for disease management, to gather information relevant to people's health-circumstances
Rhebergen et al. (2012) [111]	Quasi-experimental study (Intervention; quantitative)	Online network ArboAntwoord, network approx. 80 OSH experts	To assess the rate of correct, evidence-based answers to Occupational Safety & Health (OSH) questions in a group of workers who used an online network of OSH experts (intervention group) compared with a group of workers who used common information sources (control group)	109 employees participated who were enrolled to a course for OSH supervisor<br>2 groups:<br>1.Intervention (n=47; ArboAntwoord)<br>2.Control (n=62; usual sites ,e.g. Google)	2 questions from a pool of 16 standardized OSH questions, which were assessed using 16 standardized correct model answers	Of the 94 answers given by participants in the intervention group, 58 were correct (62%), compared with 24 of the 124 answers (19%) in the control group - difference between the 2 groups was significant ($P < 0.05$)<br><br>No differences identified between workers who provided correct answers & workers who did not on how they experienced the credibility, completeness, & applicability of the information found ($P > .05$)	Workers are often unable to find correct answers to OSH questions when using common information sources<br><br>Expert advice provided through an online expert network can be effective for this purpose	Online health-focused networks can be effective in helping workers find correct answers to health & safety questions

Abbreviations:
SNS= Social Network Site
SN = Social Network
