# Supplementary material for: A New Dimension of Health Care: Systematic Review of the Uses, Benefits, and Limitations of Social Media for Health Communication
Source: J Med Internet Res. 2013 Apr 23;15(4):e85. doi: 10.2196/jmir.1933 (PMC3636326; doi:10.2196/jmir.1933)
Supplement: Supplementary file 3 [file jmir_v15i4e85_app3.pdf]

Multimedia Appendix 2. Study quality scores using Downs and Black scale: checklist for measuring study quality (n=50)

Study	1	2	3	4	5	6	7	8	9	10	11	12	13	14	15	16	17	18	19	20	21	22	23	24	25	26	27	Quality Score
Clauson et al. (2008) [17]	1	1	1	0	0	2	0	0	0	0	1	1	0	0	0	0	0	1	0	1	0	0	0	0	0	0	1	10
Kovic et al. (2008) [19]	1	1	1	0	0	2	0	0	0	2	0	0	0	0	0	0	0	1	0	1	0	0	0	0	1	0	0	10
Timpka et al. (2008) [23]	1	1	1	0	0	2	0	0	0	0	0	0	0	0	0	0	0	0	0	1	0	0	0	0	0	0	0	6
Chou et al. (2009)[25]	1	1	1	0	0	2	2	0	0	2	1	1	0	0	0	0	0	1	0	1	0	0	0	0	1	0	0	14
Hughes et al. (2009) [29]	1	1	1	0	1	2	0	2	1	0	1	0	0	0	0	0	0	0	0	1	0	0	0	0	1	1	0	13
Jennings et al. (2009) [30]	1	1	1	1	0	1	2	0	0	2	0	0	0	0	0	0	0	1	0	1	0	0	0	0	1	0	0	12
Lupianez-Villanueva et al. (2009) [33]	1	1	1	0	1	2	0	0	0	0	1	1	0	0	0	0	0	0	0	1	0	0	0	0	0	0	0	9
Moreno et al. (2009a)[35]	1	1	1	1	1	2	2	1	2	2	0	0	1	0	0	0	0	1	1	1	1	1	1	0	0	0	0	21
Takahashi et al. (2009)[38]	1	1	1	0	0	2	2	2	0	2	1	1	0	0	0	0	0	1	0	1	0	0	0	0	0	0	0	15
Avery et al. (2010)[42]	1	1	1	0	0	2	2	0	0	2	0	0	0	0	0	0	0	1	0	1	0	0	0	0	0	0	0	11
Chew & Eysenbach (2010) [44]	1	1	1	0	0	2	2	0	0	2	1	1	0	0	0	0	0	1	0	1	0	0	0	0	1	0	1	15
Cobb et al. (2010)[45]	1	1	1	0	0	2	2	0	0	2	1	1	0	0	0	0	0	1	0	1	0	0	0	0	1	0	1	15
Colineau &	1	1	1	0	0	2	0	0	0	0	0	0	0	0	0	0	0	0	0	0	0	0	0	0	1	0	0	6

Paris (2010) [46]																												
Hu & Sundar (2010) [51]	1	1	1	0	0	2	2	0	0	2	0	0	0	0	0	0	0	1	0	1	0	0	0	0	0	0	0	11
Hwang et al. (2010) [52]	1	1	1	0	0	2	2	0	2	2	0	0	0	0	0	0	0	1	0	1	0	0	0	0	1	1	1	16
Kim & Kwon (2010) [53]	1	1	1	0	1	2	2	0	0	2	0	0	0	0	0	0	0	1	0	1	0	0	0	0	1	0	0	13
Kontos et al. (2010) [54]	1	1	1	0	1	2	2	0	0	2	0	0	0	0	0	0	0	1	0	1	0	0	0	0	1	0	1	14
Lariscy et al. (2010) [56]	1	1	1	0	0	2	2	0	0	2	0	0	0	0	0	0	0	1	0	1	0	0	0	0	0	0	0	11
Lo et al. (2010) [57]	1	1	1	0	0	2	0	0	0	2	0	0	0	0	1	0	0	1	0	1	0	0	0	0	0	0	1	11
Rice et al. (2010) [60]	1	1	1	0	0	2	2	0	0	2	0	0	0	0	0	0	0	1	0	1	0	0	0	0	0	0	0	11
Wicks et al. (2010) [65]	1	1	1	0	0	2	2	0	0	2	1	1	0	0	0	0	0	1	0	1	0	0	0	0	1	0	1	15
Adrie et al. (2011) [66]	1	1	1	0	0	2	2	0	0	0	0	0	0	0	0	0	0	0	0	1	0	0	0	0	0	0	0	8
Baptist et al. (2011) [67]	1	1	1	0	0	2	2	0	0	2	0	0	0	0	0	0	0	1	0	1	0	0	0	0	1	0	1	13
Bosslet et al. (2011) [69]	1	1	1	0	0	2	2	0	0	2	1	1	0	0	0	0	0	1	0	1	0	0	0	0	0	0	1	14
Dowdell et al. (2011) [72]	1	1	1	0	0	2	1	0	0	0	0	0	0	0	0	0	0	0	0	1	0	0	0	0	0	0	0	7
Frimming et al. (2011) [76]	1	1	1	1	0	2	0	0	0	0	0	0	0	0	0	0	0	1	1	1	0	0	0	0	0	0	0	9
Garcia-Romero et al. (2011) [79]	1	0	1	0	0	1	0	0	0	0	0	0	0	0	0	0	0	0	0	0	0	0	0	0	0	0	0	3
Hanson et al.	1	1	1	0	0	2	0	0	0	2	0	0	0	0	0	0	0	1	0	1	0	0	0	0	1	0	1	11

(2011) [80]																												
Jent et al. (2011) [82]	1	1	1	0	0	2	2	0	0	2	0	0	0	0	0	0	0	1	0	1	0	0	0	0	1	0	1	13
Kadry et al. (2011) [83]	1	1	1	0	0	2	2	0	0	2	0	0	0	0	0	0	0	1	0	1	0	0	0	0	1	0	1	13
Kishimoto & Fukushmima (2011) [84]	1	1	1	0	0	2	2	0	0	2	0	0	0	0	0	0	0	1	0	1	0	0	0	0	1	0	1	13
Kukreja et al. (2011) [85]	1	1	1	0	0	2	1	0	0	0	0	0	0	0	0	0	0	0	0	1	0	0	0	0	1	0	0	8
Lau (2011) [87]	1	1	1	0	0	2	0	0	0	2	0	0	0	0	0	0	0	1	0	1	0	0	0	0	0	0	0	9
Lord et al. (2011) [89]	1	1	1	0	0	2	2	0	0	2	0	0	0	0	0	0	0	1	0	1	0	0	0	0	1	0	0	12
Morturu & Liu (2011) [90]	1	1	1	0	0	2	1	0	0	2	1	0	0	0	0	0	0	1	0	1	0	0	0	0	0	0	0	11
O’Dea & Campbell (2011) [91]	1	1	1	0	0	2	0	0	0	0	2	0	0	0	0	0	0	1	0	1	0	0	0	0	1	0	0	10
Omurtag et al. (2011) [92]	1	1	1	0	0	2	0	0	0	0	2	1	1	0	0	0	0	1	0	1	0	0	0	0	1	0	0	12
Rajagopalan et al. (2011) [93]	1	1	1	0	0	2	2	0	0	2	0	0	0	0	0	0	0	1	0	1	0	0	0	0	0	0	0	11
Ralph et al. (2011) [94]	1	1	1	0	0	2	2	0	0	2	1	1	0	0	0	0	0	1	0	1	0	0	0	0	1	0	0	14
Selkie et al. (2011) [97]	1	1	1	0	0	2	0	0	0	0	0	0	0	0	0	0	0	0	0	1	0	0	0	0	0	0	0	6
Setoyama et al. (2011) [98]	1	1	1	0	0	2	2	0	0	2	0	0	0	0	0	0	0	1	0	1	0	0	0	0	1	0	1	13
Signorini et al.	1	1	1	0	0	2	2	0	0	0	1	1	0	0	0	0	0	0	0	1	0	0	0	0	0	0	0	10

(2011) [101]																												
Turner – McGrievy & Tate (2011) [102]	1	1	1	1	1	2	2	1	2	2	0	0	0	1	0	0	1	1	1	1	1	1	1	1	1	1	1	26
Usher et al. (2011) [103]	1	1	1	0	0	2	2	0	0	2	1	1	0	0	0	0	0	1	0	1	0	0	0	0	0	0	0	13
Van Uden-Kraan (2011) [104]	1	1	1	0	0	2	2	0	0	2	1	0	0	0	0	0	0	1	0	1	0	0	0	0	1	0	1	14
Weitzman et al. (2011) [106]	1	1	1	0	0	2	2	0	0	2	0	0	0	0	0	0	0	1	0	1	0	0	0	0	0	0	1	12
Young & Rice (2011) [107]	1	1	1	0	0	2	2	0	0	2	0	0	0	0	0	0	0	1	0	1	0	0	0	0	1	0	0	12
Fernandez-Luque et al. (2012) [108]	1	1	1	0	0	2	2	0	0	2	0	0	0	0	0	0	0	1	0	1	0	0	0	0	1	0	1	13
O'Grady et al. (2012) [110]	1	1	1	0	0	2	0	0	0	0	0	0	0	0	0	0	0	0	0	1	0	0	0	0	0	0	0	6
Rhebergen et al. (2012) [111]	1	1	1	1	1	2	2	0	0	2	1	1	1	1	1	0	0	1	1	1	1	1	0	0	1	0	0	22

Key:
Reporting: "Yes=1," "No=0"

1. Is the hypothesis /aim /objective of the study clearly described?
2. Are the main outcomes to be measured clearly described in the Introduction or Methods section?
3. Are the characteristics of the patients / samples included in the study clearly described?
4. Are the interventions of interest clearly described?
5. Are the distributions of principal confounders in each group of subjects to be compared clearly described?

“Yes=2,” “Partially=1,” “No=0”

6. Are the main findings of the study clearly described?
7. Does the study provide estimates of the random variability in the data for the main outcomes?
8. Have all important adverse events that may be a consequence of the intervention been reported?
9. Have the characteristics of patients lost to follow-up been described?
10. Have actual probability values been reported (e.g., 0.035 rather than <0.05) for the main outcomes except where the probability value is less than 0.001?

External validity: “Yes=1,” “No=0,” “Unable to determine=0”
11. Were the subjects asked to participate in the study representative of the entire population from which they were recruited?
12. Were those subjects who were prepared to participate representative of the entire population from which they were recruited?
13. Were the staff, places, and facilities where the patients were treated, representative of the treatment the majority of patients receive?

Internal validity - bias: “Yes=1,” “No=0,” “Unable to determine=0”
14. Was an attempt made to blind study subjects to the intervention they have received?
15. Was an attempt made to blind those measuring the main outcomes of the intervention?
16. If any of the results of the study were based on “data dredging” was this made clear?
17. In trials and cohort studies, do the analyses adjust for different lengths of follow-up of patients, or in case-control studies, is the time period between the intervention and outcome the same for cases and controls?
18. Were the statistical tests used to assess the main outcomes appropriate?
19. Was compliance with the intervention/s reliable?
20.Were the main outcome measures used accurate (valid and reliable)?

Internal validity - confounding (selection bias): “Yes=1,” “No=0,” “Unable to determine=0”
21. Were the patients in different intervention groups (trials and cohort studies) or were the cases and controls (case-control studies) recruited from the same population?
22. Were study subjects in different intervention groups (trials and cohort studies) or were the cases and controls (case-control studies) recruited over the same period of time?
23.Were study subjects randomized to intervention groups?
24.Was the randomized intervention assignment concealed from both patients and health care staff until recruitment was complete and irrevocable?

25. Was there adequate adjustment for confounding in the analyses from which the main findings were drawn?
26. Were losses of patients to follow-up taken into account?

Power
27. Did the study have sufficient power to detect a clinically important effect where the probability value for a difference being due to chance is less than 5%? Sample sizes have been calculated to detect a difference of x% and y%.
Size of smallest intervention group

1. A 1<n1 0
2. B n1-n2 1
3. C n3-n4 2
4. D n5-n6 3
5. E n7-n8 4
6. F n8+ 5
